# T-cell stimulating vaccines empower CD3 bispecific antibody therapy in solid tumors

**DOI:** 10.1038/s41467-023-44308-6

**Published:** 2024-01-02

**Authors:** Jim Middelburg, Marjolein Sluijter, Gaby Schaap, Büşra Göynük, Katy Lloyd, Vitalijs Ovcinnikovs, Gijs G. Zom, Renoud J. Marijnissen, Christianne Groeneveldt, Lisa Griffioen, Gerwin G. W. Sandker, Sandra Heskamp, Sjoerd H. van der Burg, Tsolere Arakelian, Ferry Ossendorp, Ramon Arens, Janine Schuurman, Kristel Kemper, Thorbald van Hall

**Affiliations:** 1 Department of Medical Oncology, Oncode Institute, Leiden University Medical Center, Leiden, the Netherlands; 2 Genmab, Utrecht, the Netherlands; 3 Department of Medical Imaging, Radboud Institute for Molecular Life Sciences, Nijmegen, the Netherlands; 4 Department of Immunology, Leiden University Medical Center, Leiden, the Netherlands

**Keywords:** Cancer immunotherapy, Immunization, Peptide vaccines

## Abstract

CD3 bispecific antibody (CD3 bsAb) therapy is clinically approved for refractory hematological malignancies, but responses in solid tumors have been limited so far. One of the main hurdles in solid tumors is the lack of sufficient T-cell infiltrate. Here, we show that pre-treatment vaccination, even when composed of tumor-unrelated antigens, induces CXCR3-mediated T-cell influx in immunologically ‘cold’ tumor models in male mice. In the absence of CD3 bsAb, the infiltrate is confined to the tumor invasive margin, whereas subsequent CD3 bsAb administration induces infiltration of activated effector CD8 T cells into the tumor cell nests. This combination therapy installs a broadly inflamed Th1-type tumor microenvironment, resulting in effective tumor eradication. Multiple vaccination formulations, including synthetic long peptides and viruses, empower CD3 bsAb therapy. Our results imply that eliciting tumor infiltration with vaccine-induced tumor-(un)related T cells can greatly improve the efficacy of CD3 bsAbs in solid tumors.

## Abstract (web summary)

CD3 bispecific antibodies (bsAbs) have demonstrated promising clinical responses in hematological malignancies but clinical benefit in solid tumors has been limited. Here the authors report that pre-treatment vaccination promotes the infiltration of tumor-(un)related effector CD8 T cells, improving the efficacy of CD3 bsAbs in solid tumors.

## Introduction

CD3 bispecific antibody (bsAb) therapy is becoming an increasingly prevalent modality in the field of cancer treatment. These bsAbs simultaneously bind to a tumor-associated surface antigen with one arm and to CD3 on the T-cell surface with the other, resulting in T-cell activation and subsequent tumor cell killing^1^. The benefit of this therapy is the independence of T-cell receptor specificity, meaning that all T cells within the tumor microenvironment (TME) could act as potential effector cells^2^.

CD3 bsAbs have demonstrated strong clinical responses in hematological malignancies, such as refractory B-cell lymphomas and lymphoblastic leukemias, and are now available in the treatment arsenal of oncologists^3,4^. Except for the recent success of Tebentafusp, targeting the gp100 peptide in HLA-A*02:01 in uveal melanoma patients^5^, the benefit of CD3 bsAb therapy in solid tumors is limited, as several hurdles appear to be present^6^. In contrast to leukemias that reside in the circulation and are surrounded by T cells, solid malignancies are often poorly infiltrated with T cells. This is most prominent in immunologically ‘cold’ tumors, which hardly exhibit any T-cell infiltrate^7^. Additionally, the TME of solid cancers is generally hostile and immunosuppressive for cytotoxic T cells, due to factors such as low pH, hypoxia, nutrient deprivation and high concentration of oxygen radicals, which can all hamper the effector functions of T cells^6,8^.

Here, we aim to overcome the hurdles of low T-cell infiltration and the hypofunctionality of existing tumor-resident T cells via the administration of vaccines, using immunologically ‘cold’ syngeneic mouse tumor models and a fully murine CD3xTRP1 bsAb. Unrelated vaccines, which do not contain tumor antigens, induce activation and influx of peripheral T cells into the tumor and act as critical effector cells for CD3 bsAb engagement. Our results demonstrate a widely applicable strategy to enhance the efficacy of CD3 bsAb treatment in solid tumors.

## Results

### Enhancing T-cell infiltration in B16F10 melanoma improves CD3 bsAb therapy

**Fig. 1:**
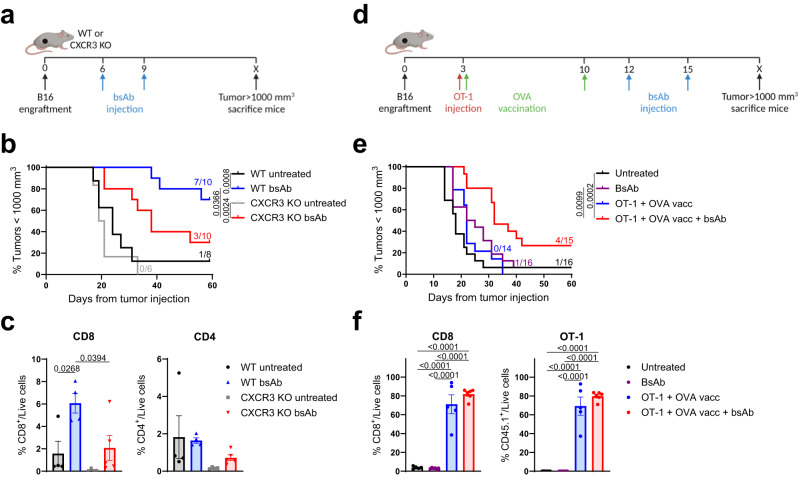
T-cell infiltration improves CD3xTRP1 bsAb therapy in B16F10 melanoma. CD3xTRP1 treatment in WT or CXCR3 KO mice bearing B16F10 mouse melanoma tumors. **a** Treatment schedule. **b** Kaplan-Meier survival graph for indicated groups. Numbers indicate the fraction of mice alive at the end of the experiment. **c** Frequencies of intratumoral CD8 and CD4 T cells 4 days after CD3xTRP1 treatment. CD3xTRP1 treatment in combination with adoptive transfer of OT-1 cells and OVA peptide vaccination in mice bearing B16F10 tumors. **d** Treatment schedule. **e** Kaplan-Meier survival graph for indicated groups. Numbers indicate the fraction of mice alive at the end of the experiment. **f** Frequencies of intratumoral total CD8 and OT-1 T cells in B16F10 tumors at day 19, according to the schedule in Supplementary Fig. 2b. Data represented as mean ± SEM (**c**, **f**). **c** (WT untreated (*n* = 4), WT bsAb (*n* = 4), CXCR3 KO untreated (*n* = 4), CXCR3 KO bsAb (*n* = 5)), **f** (Untreated (*n* = 5), BsAb (*n* = 6), OT-1 + OVA vacc (*n* = 5), OT-1 + OVA vacc + bsAb (*n* = 6)). Statistics of survival were calculated using Mantel-Cox Log-Rank tests (**b,** **e**) and T-cell frequencies were compared using one-way ANOVA tests and Tukey’s post-hoc analyses comparing all groups (**c**, **f**). Source data are provided as a Source Data file.

We previously reported therapeutic efficacy of a TRP1-targeting Fc-inert CD3 bsAb (CD3xTRP1) in an immunocompetent syngeneic B16F10 melanoma mouse model, which is described as immunologically “cold”^9,10^. To investigate the dependency of this therapy on T-cell influx, we utilized knock-out (KO) mice lacking the C-X-C motif chemokine receptor 3 (CXCR3), a key chemokine receptor for T-cell trafficking towards tumors^11^. We inoculated wild-type (WT) or CXCR3 KO mice with B16F10 tumor cells and administered CD3xTRP1 at an early timepoint (day six and nine; Fig. 1a). The antitumor activity of CD3xTRP1 in CXCR3 KO mice was significantly impaired compared to their WT counterparts, indicating that therapeutic efficacy was at least partially dependent on T cells’ ability to home towards the tumor (Fig. 1b and Supplementary Fig. 1a, b). To understand the mechanism of action, we studied the TME in WT and CXCR3 KO mice four days after CD3xTRP1 treatment using 46-parameter spectral flow cytometry and OMIQ cluster analysis (Supplementary Fig. 1c–e). Following CD3xTRP1 therapy, a significant reduction in CD8 T-cell infiltration was observed in tumors of CXCR3 KO mice (Fig. 1c). Importantly, infiltrated CD8 T cells expressed similar levels of granzyme-B (GzmB) in both WT and CXCR3 KO mice, suggesting equal cytotoxic potential (Supplementary Fig. 1f). Furthermore, despite a slight trend, no significant reduction in intratumoral CD4 T and NK cells, as well as no differences in the frequency and phenotype of myeloid cells, were detected (Fig. 1c and Supplementary Fig. 1f, g). Together, these results imply that reduced T-cell infiltration into tumors was responsible for the decreased therapeutic effect of CD3xTRP1 in CXCR3 KO mice, suggesting that homing of T cells towards the tumor is necessary for efficacious CD3 bsAb therapy in this “cold” tumor model.

We hypothesized that therapeutically increasing the frequency of intratumoral T cells could improve the outcome of CD3xTRP1 treatment. As CD3 bsAbs activate T cells independent of their TCR specificity, we used adoptive cell transfer of tumor-nonspecific OT-1 T cells (ACT) as a universal approach, to examine the effect of increasing intratumoral T-cell numbers on CD3 bsAb therapy. Naïve OT-1 CD8 T cells, recognizing the chicken ovalbumin (OVA) epitope, were adoptively transferred into B16F10-bearing mice and subjected to a prime-boost vaccination regimen with a synthetic long peptide (SLP) containing the cognate CD8-restricted OVA epitope, the toll-like receptor (TLR)7/8 agonist imiquimod as adjuvant, and supplemented by IL-2, followed by administration of CD3xTRP1 (Fig. 1d). To better reflect the clinical situation in “cold” tumors, where CD3 bsAb monotherapy has limited efficacy, a late time point (day twelve and fifteen) was chosen for the CD3xTRP1 administration. As expected, no effect of CD3 bsAb monotherapy was observed due to the late administration of CD3xTRP1 (Fig. 1e and Supplementary Fig. 2a). However, combining CD3xTRP1 with tumor-nonspecific OT-1 T cell transfer and OVA SLP vaccination significantly delayed tumor outgrowth, improved the survival rate and increased the percentage of long-term survivors. TME analysis by flow cytometry two days after bsAb administration revealed that OVA peptide vaccination induced a strong increase in intratumoral OT-1 T cells, despite the absence of their cognate antigen in the tumor (Fig. 1f and Supplementary Fig. 2b, c). These results indicate that increased infiltration into the tumor, in combination with CD3 bsAb, augments survival irrespective of T-cell specificity.

### Vaccination induces intratumoral infiltration of T cells that are locally activated by CD3xTRP1

**Fig. 2:**
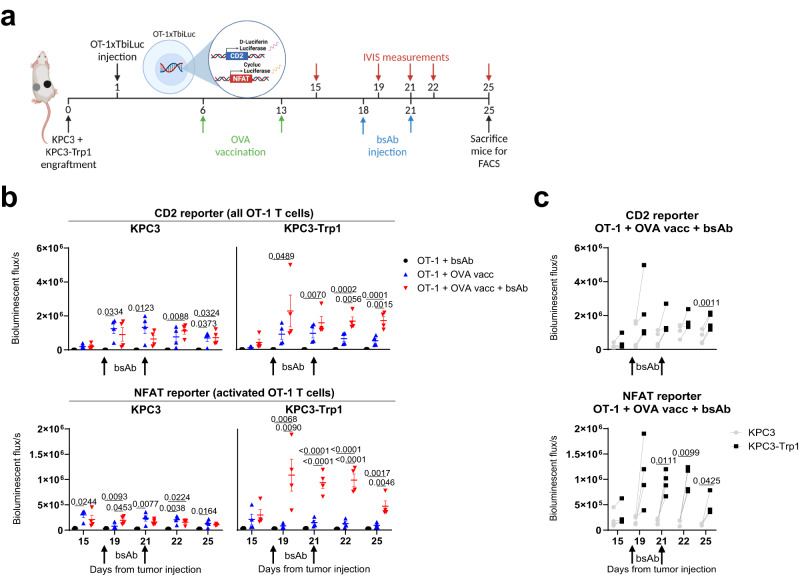
In vivo imaging of OT-1 T cells after OVA vaccination and CD3xTRP1. **a** Treatment schedule for mice inoculated with KPC3 and KPC3-TRP1 pancreatic tumor cells on distinct flanks. Transgenic dual luciferase reporter OT-1 T cells (OT-1xTbiLuc) were transferred to tumor-bearing mice, followed by OVA peptide vaccination and CD3xTRP1 treatment. The presence and activity of OT-1 T cells were monitored by bioluminescence (IVIS imaging). **b** Quantification of bioluminescent flux in KPC3 and KPC3-TRP1 tumors. CD2 reporter bioluminescent flux reflects the total amount of OT-1 T cells within the tumors and NFAT reporter flux reflects the activation of OT-1 T cells within the tumors. **c** Quantification of bioluminescent flux in KPC3 and KPC3-TRP1 tumors. Paired data points indicate tumors within the same mouse. Data represented as mean ± SEM of *n* = 4. Significance was calculated using one-way ANOVA and Tukey’s post-hoc tests comparing all groups (**b**), or two-tailed paired *t*-tests (**c**). Source data are provided as a Source Data file.

Next, we investigated the dynamics of T-cell distribution and activation for the combination treatment of OT-1 ACT, OVA vaccination and CD3xTRP1 in more detail. To visualize these dynamics in real time, we used OT-1 CD8 T cells derived from transgenic OT-1xTbiLuc mice, that express two different types of luciferase. Click-beetle luciferase, under control of the constitutively expressed CD2 promotor, was used to detect the presence of OT-1 cells by converting D-Luciferin substrate. Firefly luciferase, under control of the activation-induced NFAT promoter was used to detect the activation of OT-1 cells by converting CycLuc1 substrate^12^. In this way, T-cell distribution and activation could be monitored simultaneously over time using a bioluminescence in vivo imaging system (IVIS). Moreover, to discern systemic effects from CD3 bsAb-mediated intratumoral effects, we investigated these dynamics in mice inoculated with TRP1-positive and -negative pancreatic KPC3 tumor cells at opposite flanks (Fig. 2a). Similar to the B16F10 model, KPC3 tumors also represent a “cold” TME^13^. We observed limited tumor infiltration by naïve OT-1 cells, even when high cell numbers were transferred, with most homing towards lymphoid organs (Fig. 2b and Supplementary Fig. 3a, b). However, activation of OT-1 cells by OVA SLP vaccination resulted in a peak of infiltration into both KPC3 and KPC3-TRP1 tumors (Fig. 2b and Supplementary Fig. 3c, d), as well as into the spleen (Supplementary Fig. 3d). Addition of CD3xTRP1 to OT-1 ACT and OVA vaccination induced a strong increase in intratumoral activation and sustained presence of OT-1 cells selectively in the TRP1-positive tumors, while no CD3 bsAb-mediated effects were observed in the TRP1-negative tumors (Fig. 2b, c). Analysis of tumors by flow cytometry at day 25 confirmed the increased OT-1 infiltration and activation specifically in the TRP1-positive tumors after the triple combination therapy (Supplementary Fig. 3d). We concluded that T cells infiltrate into “cold” tumors in response to vaccination, but a further local burst of T-cell activation induced by CD3 bsAbs is dependent on tumoral expression of the target antigen.

**Fig. 3:**
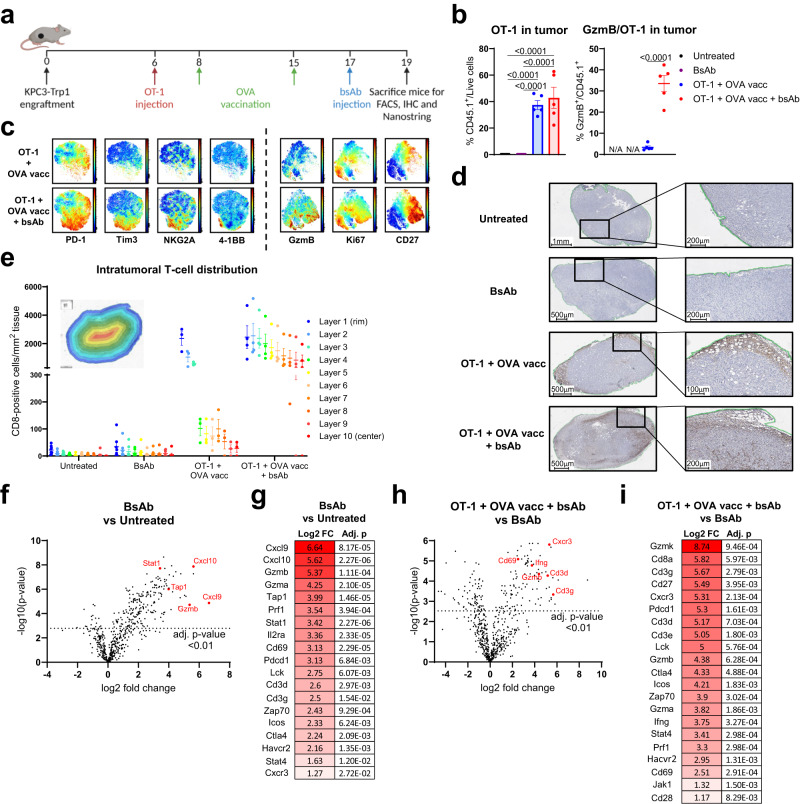
CD3xTRP1 induces activation and deep infiltration of vaccine-recruited OT-1 T-cells. **a** Treatment schedule for mice bearing KPC3-TRP1 tumors receiving OT-1 T cells, OVA vaccination and CD3xTRP1 treatment. **b** Frequencies of intratumoral OT-1 T cells and expression of GzmB by OT-1 cells in KPC3-TRP1 tumors. **c** Expression of T-cell activation markers (mean fluorescence intensity) by OT-1 T cells for indicated groups in opt-SNE plots, generated using OMIQ software. Color scale indicates expression: red, high; blue, low. Representative images (**d**) and quantification (**e**) of CD8 IHC staining of KPC3-TRP1 tumors. Color gradient indicates depth of infiltration: red, high; blue, low. Nanostring transcriptomics analysis with the PanCancer Immune Profiling Panel showing volcano plots of differentially expressed genes (**f** and **h**) and a selected list of differentially expressed genes (**g** and **i**). Data represented as mean ± SEM (**b** and **e**). **b** (Untreated (*n* = 7), BsAb (*n* = 7), OT-1 + OVA vacc (*n* = 5), OT-1 + OVA vacc + bsAb (*n* = 5)), **e** (Untreated (*n* = 8), BsAb (*n* = 7), OT-1 + OVA vacc (*n* = 3), OT-1 + OVA vacc + bsAb (*n* = 4)), **f**–**i** (Untreated (*n* = 5), BsAb (*n* = 5), OT-1 + OVA vacc + bsAb (*n* = 3)). Significance was calculated using one-way ANOVA and Tukey’s post-hoc tests comparing all groups (**b**), or unpaired two-sided *t* tests and Benjamini-Hochberg correction for multiple comparisons (**f**–**i**). Source data are provided as a Source Data file.

To expand on our IVIS imaging observations, we studied tumor infiltration and activation of OT-1 T cells in more depth using high-dimensional flow cytometry, immunohistochemistry (IHC) and Nanostring transcriptomics (Fig. 3a). In line with the in vivo imaging results, OT-1 ACT with OVA vaccination strongly increased T-cell tumor infiltration (Fig. 3b, Supplementary Fig. 4a). Adding CD3xTRP1 treatment resulted in the emergence of a population of highly activated OT-1 T cells in the tumor, reflected by a significant upregulation of intracellular GzmB expression, and high expression of multiple activation markers, such as 4-1BB, CD27, NKG2A, PD-1 and Tim3 (Fig. 3b, c). Ki-67 expression in OT-1 T cells was similar in tumors of mice receiving vaccination with or without subsequent CD3xTRP1 administration, indicating that at this time point (two days after CD3 bsAb treatment, Fig. 3c) OT-1 proliferation was induced by the vaccination and not by the CD3 bsAb. We observed similar findings in B16F10 tumors (Supplementary Fig. 4b). Subsequent treatment with CD3 bsAbs did not induce any significant differences in the activation status of OT-1 cells in the spleen, corroborating the notion that the antibody-mediated activation burst is localized to the TME (Supplementary Fig. 4c). IHC staining for CD8 confirmed the presence of T cells within the tumor after OT-1 ACT and OVA vaccination (Fig. 3d, e and Supplementary Fig. 4d, e). Importantly, these CD8 T cells were mainly present at the rim of the tumor, whereas administration of CD3xTRP1 facilitated deeper influx of CD8 T cells into the tumor centers (Fig. 3e). Transcriptomic analysis revealed a significant upregulation of pro-inflammatory genes, such as *Cxcl9*, *Cxcl10*, *Gzmb*, *Stat1* and *Tap1*, and T-cell receptor-associated transcripts (*Cd3d* and *Cd3g*) after CD3xTRP1 administration compared to untreated tumors (Fig. 3f, g and Supplementary Data 1). These genes were further upregulated in tumors treated with the triple combination therapy, illustrated by a higher expression of, for example, *Cd3d*, *Cd3g*, *Cd69*, *Cxcr3*, *Gzmb* and *Ifng* (Fig. 3h, i and Supplementary Data 2). In line with the increased presence and activation status of T cells observed by flow cytometry, we observed augmented expression of pro-inflammatory genes in mice that received the triple combination therapy compared to only OT-1 transfer and OVA vaccination (Supplementary Fig. 4f and Supplementary Data 3). When using the nSolver analysis software to stratify the differential gene expression data into cell type or pathway representations, we found an increased score for most immune cell types and related pathways when comparing the triple combination therapy to the CD3xTRP1 monotherapy. This further indicates that combining OT-1 transfer with OVA vaccination and a CD3 bsAb induces a strong and broad immune response (Supplementary Fig. 4g, h).

Together, these results show that naïve T cells, when primed by a vaccine, expand and home to all tumor sites. The addition of CD3 bsAb leads to a strong local burst of tumoricidal effector functions and deeper infiltration of CD8 T cells selectively within tumors expressing the target antigen, leading to enhanced antitumor activity.

### Biodistribution of CD3xTRP1 is not influenced by OT-1 transfer and OVA vaccination

Although the binding affinity of the CD3 arm of CD3xTRP1 is lower than the TRP1-binding arm^14,15^, the CD3 bsAb could theoretically piggyback on T cells to reach tumor sites. The combination of T-cell transfer and vaccination with CD3xTRP1 might therefore affect the biodistribution of CD3xTRP1. To evaluate this distribution in vivo, CD3xTRP1 was labeled with radioactive indium-111 and imaged using single-photon emission computerized tomography (SPECT) technology (Supplementary Fig. 5a). Analysis of the blood revealed that the majority of CD3xTRP1 was located in the cell-free serum fraction, whereas only a minor fraction was bound to cells (Supplementary Fig. 5b). This distribution ratio was comparable in all treatment groups. For the combinations with OVA vaccination, analysis of the tissues showed a tendency towards increased CD3xTRP1 distribution into CD3-rich organs, such as the spleen and lymph nodes (LNs) (Supplementary Fig. 5c, d). This was accompanied by a slightly increased accumulation of CD3xTRP1 in the KPC3 tumor, muscle and liver, which is most likely attributed to elevated amounts of T cells in these tissues as a result of the vaccination. Addition of OT-1 transfer to the OVA vaccination and CD3 bsAb treatment, a combination previously shown to induce strong infiltration of OT-1 T cells into the tumor (Fig. 3b), did not result in an increase in CD3xTRP1 accumulation, suggesting that the CD3 bsAb is not piggybacking on T cells to reach tumors.

### Influx of endogenous CD8 T cells is essential for durable treatment responses

**Fig. 4:**
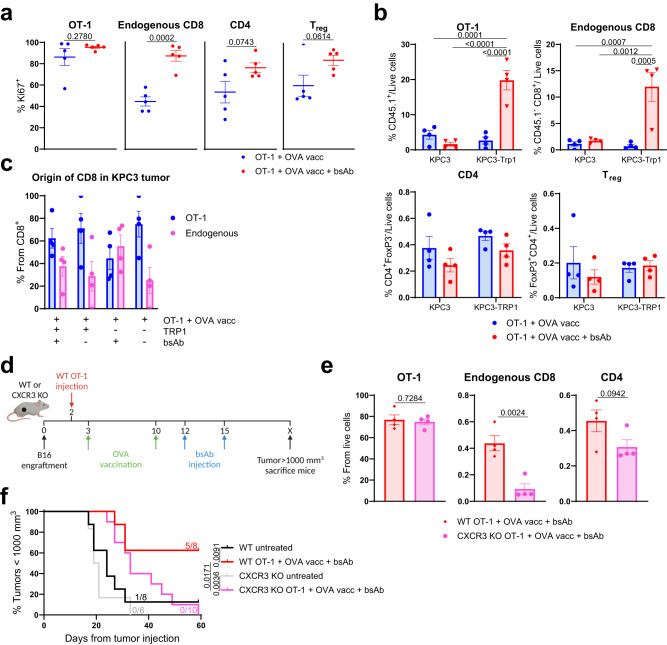
Endogenous CD8 T cells are crucial for durable tumor control by combination treatment. **a** Expression of Ki67 on T cells in KPC3-TRP1 tumors treated with OT-1, OVA SLP vaccination and CD3xTRP1, two days after CD3xTRP1 administration according to the schedule in Fig. 3a. Frequencies of intratumoral OT-1 and endogenous CD8, CD4 and regulatory T cells out of live cells (**b**), or as a population out of the total CD8 T cells (**c**), as measured by flow cytometry in mice bearing KPC3 or KPC3-TRP1 tumors treated with OT-1, OVA SLP vaccination and CD3xTRP1, 7 days after the first CD3xTRP1 administration according to the schedule in Fig. 2a. **d** Treatment schedule for WT or CXCR3 KO mice bearing B16F10 mouse melanoma receiving wild type OT-1 cells, OVA SLP vaccination and CD3xTRP1. **e** Frequencies of intratumoral T-cell populations within B16F10 tumors of WT or CXCR3 KO mice treated according to the schedule of Supplementary Fig. 6b. **f** Kaplan-Meier graph for indicated groups. Numbers indicate fractions of mice alive at the end of the experiment. Data represented as means ± SEM of *n* = 5 (**a**–**c**) or *n* = 4 (**e**). Significance was calculated using unpaired two-sided *t*-tests (**a**, **e**), one-way ANOVA and Tukey’s post-hoc tests comparing all groups (**b**) and Mantel-Cox Log-Rank tests (**f**). Source data are provided as a Source Data file.

In the experiments described above, we utilized adoptively transferred OT-1 T cells to trace their homing and activation during the combination therapy. We next investigated the contribution of endogenous T cells to the efficacy of the combination therapy, since endogenous T cells would be equally capable of responding to vaccination as OT-1 cells. We further argued that removal of the ACT could simplify the treatment regimen and increase its potential for clinical translation. Therefore, we first analyzed the proliferation and infiltration of the endogenous CD8 T-cell subset with the triple combination treatment protocol (according to Fig. 3). We observed increased Ki67 expression in the endogenous intratumoral CD8 T-cell population two days after administration of CD3xTRP1, while their frequencies were not yet enhanced (Fig. 4a and Supplementary Fig. 6a). We then studied frequencies of endogenous CD8 T cells (CD45.1^−^) in the triple combination group at a later time point (seven days after bsAb administration) and found that approximately 35% of the total intratumoral CD8 T-cell population in the KPC3-TRP1 tumors was derived from the endogenous CD8 T-cell pool (Fig. 4b, c). Influx of conventional CD4 T cells and T_regs_ was not altered, likely because the administered OVA vaccine only contained a CD8 T-cell epitope. In line with our bioluminescence analyses (Fig. 2c), CD3xTRP1 treatment induced increased frequencies of OT-1 and endogenous CD8 T cells only in the TRP1-positive and not the TRP1-negative tumors, implying that intratumoral engagement by CD3xTRP1 is required for expansion and prolonged presence of intratumoral T cells, initially recruited by vaccination (Fig. 4b).

We hypothesized that the infiltration of endogenous CD8 T cells into the tumor was contributing to the efficacy of the triple combination treatment. To study this, we excluded endogenous T-cell influx by using CXCR3 KO mice bearing B16F10 tumors and the triple combination treatment protocol: adoptively transferred WT OT-1 cells, OVA vaccination and CD3xTRP1 (Fig. 4d and Supplementary Fig. 6b). Similar frequencies of intratumoral OT-1 T cells were observed in the WT and CXCR3 KO mice, due to their WT background, but significantly lower frequencies of endogenous CD8 T cells were detected in tumors of CXCR3 KO mice (Fig. 4e and Supplementary Fig. 6b). Importantly, despite normal influx of OT-1 T cells and other immune subsets (Supplementary Fig. 6c, d), combination treatment in CXCR3 KO mice failed to induce durable responses, thereby implying that the influx of endogenous CD8 T cells was essential for the anti-tumor response (Fig. 4f and Supplementary Fig. 6e). Altogether, these results suggest that the triple combination therapy first results in strong influx and activation of OT-1 T cells in the tumor, after which endogenous CD8 T cells are recruited and mediate long-term protection to tumor outgrowth.

### Vaccination prior to CD3xTRP1 orchestrates a broad pro-inflammatory adaptive and innate immune response

**Fig. 5:**
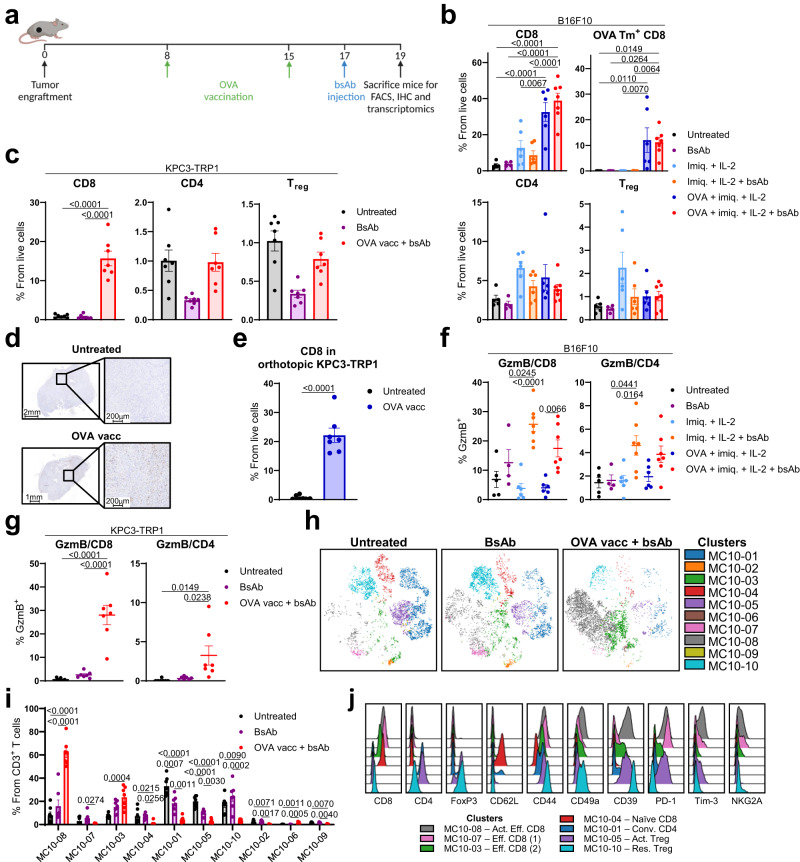
Combination of vaccination and CD3xTRP1 alters the intratumoral T-cell immune landscape. **a** Treatment schedule for mice bearing B16F10 (**b** and **f**) or KPC3-TRP1 tumors (**c**, **g**–**j**) receiving OVA vaccination and CD3xTRP1 treatment for flow cytometry, IHC and transcriptomics analyses of the TME. Frequencies of intratumoral T-cell subsets in B16F10 (**b**) or KPC3-TRP1 tumors (**c**). OVA designates the CD8 OVA peptide (**b**). CD8 T-cell infiltrate in mice bearing orthotopic KPC3-TRP1 tumors in the pancreas, treated with OVA vaccination on day 9 and 16 and sacrificed on day 20, showing CD8 IHC staining (**d**), and CD8 quantification using flow cytometry (**e**). GzmB expression of CD8 and CD4 T cells in B16F10 (**f**), or KPC3-TRP1 (**g**) tumors. OVA designates the CD8 OVA peptide (**f**). Unsupervised clustering of CD3^+^ immune cells using OMIQ software. Overlay of metaclusters on the opt-SNE results, showing 1000 CD3^+^ cells per sample for a total of seven samples per treatment group (**h**). Distribution of the metaclusters over the treatment groups (**i**). Selection of phenotypical marker expression for all metaclusters with >5% representation for any of the treatment groups (**j**). Imiq., imiquimod; Act., activated; Eff., effector; Conv., conventional; Res., resident. Data represented as mean ± SEM (**b**, **c**, **e**–**g** and **i**). **b**, **f** (Untreated (*n* = 5), BsAb (*n* = 4), Imiq. + IL-2 (*n* = 6), Imiq. + IL-2 + bsAb (*n* = 6), OVA + imiq. + IL-2 (*n* = 6), OVA + imiq. + IL-2 + bsAb (*n* = 7)), **c**, **e**, **g** and **i** (*n* = 7). Significance was calculated using one-way ANOVA and Tukey’s post-hoc tests comparing all groups (**b**, **c**, **f**, **g** and **i**) or using unpaired two-sided *t*-tests (**e**). Source data are provided as a Source Data file.

**Fig. 6:**
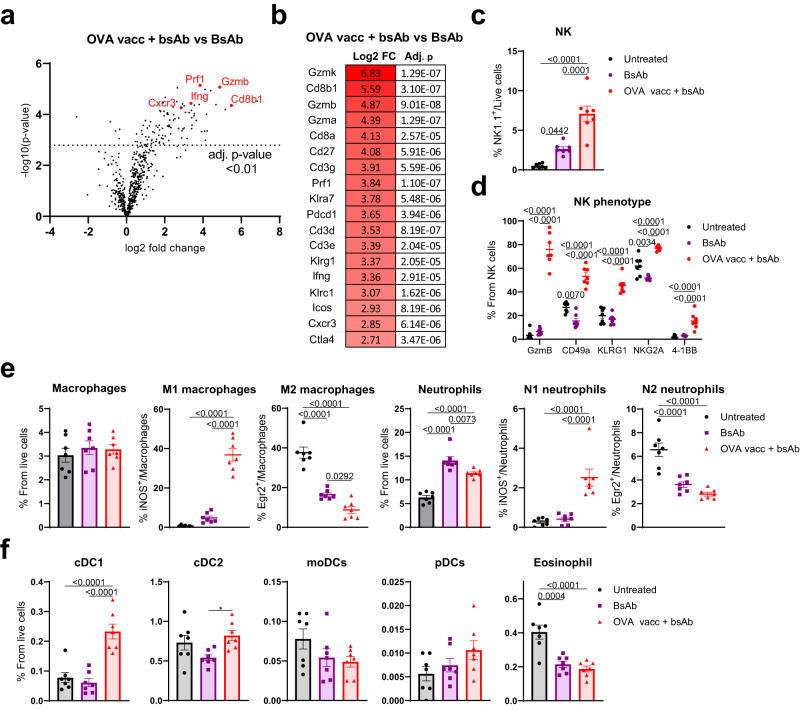
Profound pro-inflammatory imprinting of innate immune cells by combination therapy. Volcano plots of differentially expressed genes using transcriptomic analysis with PanCancer Immune Profiling Panel (**a**) and top selection of differentially expressed genes (**b**). Frequency (**c**) and phenotype (**d**) of intratumoral NK cells in KPC3-TRP1 tumors. Frequency and phenotype of macrophage and neutrophil subsets (**e**) (in which M1 macrophages and N1 neutrophils were defined as inducible nitric oxide synthase (iNOS)-positive, M2 macrophages and N2 neutrophils were defined as early growth factor receptor 2 (Egr2)-positive) and frequency of other myeloid immune cell subsets (**f**). All data are derived from KPC3-TRP1 tumors treated according to the treatment schedule depicted in Fig. 5a. Flow cytometry markers and gating strategy are displayed in Supplementary Figs. 1d and 6b. Data represented as mean ± SEM (**c**–**f**). **a**, **b** (*n* = 5), **c**–**f** (*n* = 7). Significance was calculated using unpaired two-sided *t* tests and Benjamini-Hochberg correction for multiple comparisons (**a**, **b**), or one-way ANOVA and Tukey’s post-hoc tests comparing all groups (**c**–**f**). Source data are provided as a Source Data file.

As endogenous T cells were key contributors to the treatment outcome, this prompted the removal of the OT-1 transfer from this combination approach (Fig. 5a). We then examined the extent of T-cell infiltration and activation (Fig. 5) in the OVA vaccine (containing OVA peptide, imiquimod and IL-2) and CD3xTRP1 double therapy, as well as its impact on innate immune cells (Fig. 6) in both B16F10 and KPC3-TRP1 tumor models. We found that vaccination with the CD8-restricted OVA peptide, led to infiltration of endogenous CD8 T cells, but not CD4 T cells or T_regs_, in both B16F10 (Fig. 5b) and KPC3-TRP1 (Fig. 5c) tumor models, independent of CD3 bsAb administration (Fig. 5b). As expected, a subset of these CD8 T cells were specific for the OVA epitope (Fig. 5b). Interestingly, application of the adjuvant TLR7/8 agonist imiquimod (Aldara) alone, resulted in CXCR3 expression on approximately 50% of all peripheral CD8 T cells (Supplementary Fig. 7a). However, presence of the vaccine antigen was essential for CD8 T-cell infiltration into the tumor, evident by a sharp increase in intratumoral CD8 T-cell influx with the addition of OVA peptide (Fig. 5b). To test if the observed infiltration was restricted to subcutaneous tumors, the role of OVA vaccination in intratumoral CD8 T-cell influx was also examined in an orthotopic model, where KPC3-TRP1 tumors were locally inoculated in the pancreas. In line with the subcutaneous model, tumor-nonspecific vaccination induced CD8 T-cell infiltration in the tumor, irrespective of its size (Fig. 5d, e and Supplementary Fig. 7b, c).

In both subcutaneous tumor models (B16F10 and KPC3-TRP1), we extensively studied the intratumoral T-cell subsets by high-dimensional spectral flow cytometry to understand the effect of the OVA vaccination and subsequent CD3xTRP1 treatment on T-cell activation. The double combination therapy resulted in large proportions of GzmB-positive CD8 and CD4 T cells in both tumor models (Fig. 5f, g). Interestingly, a higher percentage of GzmB-positive T cells was observed in tumors that received imiquimod and IL-2 in addition to CD3xTRP1 when compared to tumors receiving the antibody alone, suggesting that this pretreatment two days before CD3xTRP1 primed T cells for CD3 bsAb (Fig. 5f). KPC3-TRP1 tumors from three treatment groups (untreated, CD3xTRP1 alone and combination of OVA vaccination with CD3xTRP1) were subjected to in-depth unsupervised cluster analysis on the intratumoral T cells using two separate 20-marker antibody panels. The metacluster (MC) landscape revealed major changes in T-cell subsets between different treatment groups (Fig. 5h, j). In untreated tumors, CD4 T cells and T_regs_ dominated the landscape, together with a naïve-like CD62L^+^CD44^-^Tcf1^+^ CD8 T-cell population (Fig. 5i, j and Supplementary Fig. 7d MC10-01, -04, -05, -10 and Supplementary Fig. 7e–g MC10-02, -03, -09). CD3xTRP1 administration alone increased the percentages of several CD8 T-cell subsets, but the addition of vaccination heavily skewed the distribution towards activated effector CD8 T cells, illustrated by expression of markers indicating recent TCR triggering and tissue residency, like PD-1, CD69, and CD49a (Fig. 5j and Supplementary Fig. 7d MC10-03, -08 and Supplementary Fig. 7g MC10-05, -08). These findings were supported by the B16F10 model, where manual gating showed that expression of CD39, CD49a, NKG2A, and 4-1BB on CD8 T cells was induced by the combination therapy with an important role for imiquimod and IL-2 (Supplementary Fig. 7h). Altogether, these findings show that vaccination induces strong influx of CD8 T cells into the tumor and that vaccination together with CD3 bsAb provides strong activation of all T-cell subsets in the TME. The combination therapy thereby strongly alters the T-cell landscape in the tumor.

Transcriptomic analysis confirmed our observations made by flow cytometry, showing increased expression of T-cell (co)receptor genes *Cd8b1*, *Cd8a*, *Cd3g*, *Cd3d,* and *Cd3e* and T-cell effector genes, such as *Gzmk, Gzmb, Ifng* and *Prf1*, in tumors treated with OVA vaccination and CD3xTRP1 compared to antibody alone (Fig. 6a, b and Supplementary Data 4). Interestingly, these changes in gene expression coincided with high deconvolution scores for multiple immune cell types and upregulation of pathways involved in active immune responses (Supplementary Fig. 8a, b). Due to these findings, we hypothesized that the double combination therapy could also alter the innate immune landscape in the TME. Indeed, increased influx of NK cells was observed upon combination treatment when compared to antibody alone, which was characterized by high expression of GzmB, CD49a, and KLRG1 (Fig. 6c, d and Supplementary Fig. 8c, d). Furthermore, macrophage and neutrophil phenotypes shifted from immunosuppressive M2-type and N2-type, found in untreated or antibody alone groups, to predominantly pro-inflammatory M1-type macrophages and N1-type neutrophils in the combination therapy-treated tumors (Fig. 6e and Supplementary Fig. 8e–g). Furthermore, combination treatment increased the frequency of type 1 conventional dendritic cells (cDC1) in KPC3-TRP1 tumors (Fig. 6f), a cell type which is associated with increased cross-presentation and a pro-inflammatory TME^16^. However, this was not observed in the B16F10 model (Supplementary Fig. 8h). Importantly, alterations in the innate immune cell compartment did not require the presence of OVA peptide, but mainly depended on the combination of imiquimod, IL-2, and CD3xTRP1 (Supplementary Fig. 8c–g), or, in the case of most activation markers on NK cells, on imiquimod and IL-2 alone (Supplementary Fig. 8d), demonstrating the importance of the adjuvant.

Altogether, these data demonstrate that in contrast to CD3 bsAb monotherapy, combination of tumor-nonspecific vaccination with CD3 bsAb induces a coordinated and profound immune response, encompassing both adaptive and innate compartments, with polarization towards a pro-inflammatory TME.

### Several vaccines enhance the anti-tumor activity of CD3 bsAb therapy

**Fig. 7:**
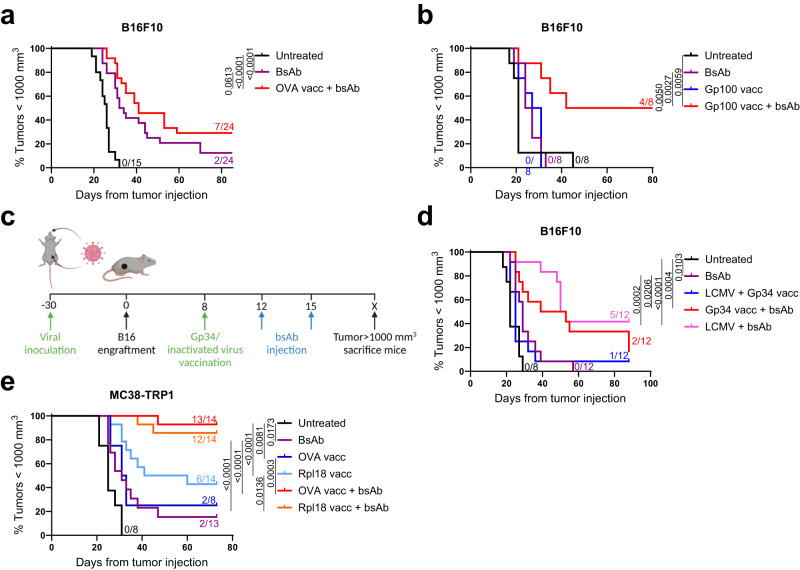
Several vaccination approaches enhance CD3xTRP1 therapy. Kaplan-Meier curves for indicated groups of B16F10-bearing mice receiving OVA vaccination (**a**), or Gp100 vaccination (**b**) according to the treatment schedule described in Fig. 1d. Numbers indicate fractions of surviving mice at the end of the experiment. **c** Treatment schedule for mice pre-inoculated with LCMV-Armstrong (for Fig. 7d) or HKx31 influenza virus (for Supplementary Fig. 10), followed by B16F10 inoculation. Indicated are administration of LCMV Gp34 peptide vaccination (for Fig. 7d), or inactivated HKx31 influenza virus (for Supplementary Fig. 10) and CD3xTRP1. **d** Kaplan-Meier curves for indicated groups receiving LCMV/Gp34 treatments. Numbers indicate fractions of surviving mice at the end of the experiment. **e** Kaplan-Meier curves for indicated groups of MC38-TRP1-bearing mice receiving OVA or Rpl18 vaccination in combination with CD3xTRP1 according to the treatment schedule in Fig. 1d. Numbers indicate fractions of surviving mice at the end of the experiment. Significance was calculated using Mantel-Cox Log-Rank tests (**a**, **b**, **d**, **e**). Source data are provided as a Source Data file.

Next, we investigated whether the profound TME remodeling translated into better antitumor activity and survival. We hypothesized that this combination strategy could be applied using various tumor-specific and nonspecific antigens and vaccine formulations. We, therefore tested several different vaccination strategies for their ability to potentiate CD3 bsAb therapy. As vaccines, we used six different antigens delivered in either the SLP or virus format, as surrogate for viral vector-based vaccinations. The tumor-nonspecific OVA SLP and the tumor-specific altered-self SLP Gp100^17^ were both adjuvanted with imiquimod and supplemented with IL-2, and the lymphocytic choriomeningitis virus (LCMV)-derived tumor-nonspecific Gp34 SLP^18^ was supplemented with TLR9 agonist CpG as adjuvant (Fig. 7a–d). Treatment with these three peptide vaccines prior to CD3xTRP1 administration delayed tumor outgrowth and increased survival, irrespective if the vaccine contained a tumor-specific or -nonspecific antigen (Fig. 7a–d and Supplementary Fig. 9a–d). Importantly, combination of imiquimod and IL-2 without peptide and CD3 bsAb did not result in better survival outcomes compared to CD3xTRP1 alone, indicating that addition of the vaccine peptide is required for the anti-tumor activity (Supplementary Fig. 9e, f). Next, LCMV-Armstrong virus inoculation was used as a model for vector-based vaccinations. This virus replicates in mice and has been shown to induce long-lasting effector T cells, despite its swift clearance^19,20^. Indeed, LCMV Gp33-specific CD8 T cells with an effector memory phenotype were observed up to 49 days after LCMV infection (Supplementary Fig. 9a, g). Importantly, combination of CD3xTRP1 with LCMV inoculation significantly enhanced long-term survival of mice compared to antibody treatment alone (Fig. 7d). Similar survival data were obtained using the HKx31 influenza virus inoculum (Supplementary Fig. 10a, b)^21^. Analysis of peripheral blood revealed an induction of T-cell responses after HKx31 infection, which were further boosted by administration of inactivated HKx31 virus (Supplementary Fig. 10c, d).

Finally, the efficacy of the combination treatment was tested in a more immunogenic tumor model. We used the colorectal MC38 cell line, transfected to express the surface protein TRP1. Importantly, the combination of CD3xTRP1 with tumor-nonspecific OVA, as well as the tumor-specific neoantigen Rpl18 SLP^22^ vaccines adjuvanted with CpG, both resulted in complete antitumor responses in almost all mice (Fig. 7e). Altogether, these results show that CD3 bsAb therapy for solid cancers can be transformed into an effective treatment by recruiting activated CD8 T cells via multiple tumor-specific and non-specific vaccine modalities.

## Discussion

In this study, we achieved potentiation of a CD3 bsAb, targeting the clinically relevant TRP1 antigen (NCT04551352), via pretreatment with vaccination strategies that induce T-cell responses. As a consequence of systemic vaccine-mediated T-cell activation, tumor-nonspecific vaccines induced effective homing of peripheral CD8 T cells towards the rim of the tumor. Subsequent administration of CD3xTRP1 further activated these T cells locally in the tumor, thereby differentiating them into potent effectors and facilitating deep infiltration into the tumor center. The combination of tumor-nonspecific vaccines with CD3 bsAb furthermore increased the frequency of activated NK cells, cross-presenting cDC1s, pro-inflammatory M1-like macrophages, and N1-like neutrophils within the tumor, thereby generating a broadly inflamed TME. Crucially, this combination treatment resulted in delayed tumor outgrowth and improved survival for multiple vaccination strategies.

Whether effective CD3 bsAb therapy depends on baseline T-cell infiltration levels, or on-treatment influx of T cells is still under debate. Several studies have shown that T-cell influx is essential for survival and used either CXCR3 KO mice (as in this study) or chemokine receptor blocking antibodies, both resulting in neutralization of CD3 bsAb-induced chemokine signaling^23–25^. In contrast, Belmontes et al. reported that not on-treatment T-cell influx, but baseline T-cell numbers in the tumor were essential for CD3 bsAb antitumor activity. This study used a different approach and trapped lymphocytes in lymphoid organs with the S1PR1 agonist FTY720 (fingolimod)^26^. A recent study by You et al., also using FTY720, has highlighted that the expression level of the targeted tumor-associated antigen (TAA) dictates whether therapy efficacy depends on baseline T-cell infiltration or therapy-induced T-cell influx^27^. In their study, treatment efficacy relied more on baseline T-cell numbers in tumors expressing high levels of TAA, whereas T-cell influx was more important for tumors expressing low to moderate levels of TAA. Nevertheless, all studies agree that higher T-cell numbers within the tumor, either at baseline or on-treatment, improves CD3 bsAb treatment outcomes. Our findings differ from those reported by Belmontes et al., which may be attributed to the use of different mouse models and cell lines. We utilized a fully syngeneic model consisting of mouse tumor cell lines with relatively low expression levels of self TRP1 TAA in non-transgenic immunocompetent mice, whereas Belmontes et al. used humanized mice (huCD3ε knock-in) and murine cell lines transfected with human TAAs, which is generally associated with relatively high levels of TAA expression and increased immunogenicity^28–30^. Given the findings of You et al.^27^, it seems plausible that the differences in levels of TAA expression may account for the observed discrepancies between the two studies. Importantly, our vaccination conditioning treatment resulted in a high frequency of intratumoral CD8 T cells that were not recruited in CXCR3 KO hosts. These high intratumoral CD8 T-cell frequencies required the presence of antigen peptide, clearly indicating that priming, clonal expansion and tumor infiltration of T cells prior to antibody treatment were essential biological principles of this therapy. In addition, subsequent engagement by the CD3 bsAb was responsible for the expansion and prolonged presence of T cells in TRP1-positive tumors. It is likely that both T-cell influx and intratumoral proliferation contribute to the therapeutic benefit of our combination therapy, as was demonstrated recently in an elegant humanized mouse model, where anti-tumor activity of a CD3xCD20 bsAb relied both on intratumoral T-cell proliferation and infiltration^25^. However, the contribution of each of these mechanisms needs to be further investigated.

In addition to CD3 bsAbs, ex vivo pre-activated T cells armed with CD3 bsAb, known as “bispecific armed T cells” (BATs), are also being developed. These BATs allow for lower dosing, which translates into fewer toxicities when compared to CD3 bsAbs in clinical trials^31^. However, responses to BATs are frequently hampered by limited T-cell infiltration and persistence in the TME^32^. Therefore, treatment with BATs might also benefit from vaccine-induced T-cell recruitment, as reported herein. Interestingly, two recent studies combined another T-cell therapy, namely the chimeric antigen receptor (CAR) T cells, and in vivo vaccination with cognate CAR antigens^33,34^. The investigators observed increased engraftment, polyfunctionality and anti-tumor efficacy of the CAR T cells upon vaccination as a result of priming by antigen-presenting cells (APCs). It is plausible to think that similar responses can be induced by off-the-shelf vaccines in combination with CD3 bsAbs, thus bypassing the need to generate transgenic T cells and develop a cognate vaccine.

Recently, several studies have reported the combination of CD3 bsAbs with intratumoral administration of oncolytic viruses (OVs), capable of replicating in tumor cells^35^. In line with the results from our combination strategy, administration of OV together with CD3 bsAbs resulted in strong T-cell influx into tumors and improved survival^13,36,37^, suggesting that the mechanism of action could be similar between these two approaches. In these studies, the delivery of OVs and CD3 bsAbs was either performed separately, or the researchers engineered CD3 bsAbs into the viral genome. The power of genetically engineered OVs is the option to add cargo to the viral genome, such as T-cell sustaining cytokines or immune checkpoint blockers that will be expressed locally in the tumor^38^. The challenge is to find the correct order and timing between OV and CD3 bsAb administration, as this has been shown to be key for optimal synergy^13^. The major difference between combining CD3 bsAbs with vaccines or OVs is the route of administration. OVs are routinely administered intratumorally, thereby limiting clinical applicability to easily accessible tumors, whereas the vaccination approach is systemic, making it more applicable for a variety of tumors, irrespective of their location.

In this study, we used several vaccination approaches to induce T-cell infiltration into the tumor, resulting in improved treatment outcomes following CD3 bsAb treatment. A major benefit of this strategy is that vaccine-recruited T cells have not been subjected to the suppressive TME, unlike T cells that were residing in the tumor^8^. Additionally, use of adjuvants as part of the vaccination strategy should ensure that co-stimulation is provided by APCs, resulting in better T-cell priming and functionality. This notion is in line with the findings described previously for the combination of CAR T cells and vaccination^34^. Here, we used either Aldara (imiquimod; TLR7/8 ligand) together with IL-2, or CpG (TLR9 ligand) as adjuvants. TLR ligands are well known to activate APCs and generate a Th1 type immune response^39–42^, whereas administration of the growth factor IL-2 is described to specifically induce activation and proliferation of lymphoid cells^43^. These vaccine components might therefore enhance CD3 bsAb therapy alone, without the addition of a vaccine antigen. Indeed, we found that imiquimod and IL-2 strongly promoted the CD3 bsAb-induced activation of all T-cell subsets and infiltration of innate immune cells, which is in line with previous observations about the effects of Aldara^41^. However, imiquimod and IL-2 combined with CD3 bsAb did not increase intratumoral T-cell frequencies and only resulted in a minimal survival benefit compared to CD3 bsAb monotherapy. In contrast, addition of a CD8-restricted peptide was necessary to generate substantial CD8 T-cell influx and significant survival benefits. Our data suggest that the role of IL-2 is limited, as IL-2 was even omitted for vaccine combinations of Gp34 LCMV peptide in the B16F10 model or OVA and Rpl18 peptide in the MC38 model, which were all adjuvanted with CpG. These findings demonstrated that while the adjuvant is an essential contributor to our combination treatment, complete vaccines are needed to confer therapeutic efficacy.

While we have focused on CD8 T-cell epitopes in our vaccines, addition of CD4 epitopes might further improve treatment outcome, as cytolytic capacity of CD4 T cells has previously been shown^44^, including in CD3 bsAb therapy^10^. In this research, we have explored vaccination strategies using SLP and virus inoculation. Alternative vaccine platforms, such as mRNA, DNA and dendritic cell vaccines should therefore be addressed in future research. Clinically approved childhood and travel vaccines, both of which could also have the additional benefit of utilizing recall responses from previous immunizations, are interesting candidates for clinical translation of this concept. Additionally, cancer vaccines might be considered as they recently regained attention after demonstrating clinical efficacy^45–47^. Such cancer vaccines could provide an additional benefit of generating long-term immunity to protect against recurrences, although this would require further investigation. The main requirement for vaccine selection is the need for potent T-cell responses, whereas most childhood and travel vaccines are known for their capability to induce protective humoral responses^48^.

To summarize, we have shown that both tumor-specific and non-specific vaccination prior to CD3 bsAb administration enhances T-cell infiltration, pro-inflammatory TME polarization and improves treatment responses in immunologically ‘cold’ tumors. We believe that combining CD3 bsAbs together with vaccine modalities capable of eliciting potent T-cell responses could soon lead to a successful clinical translation of this combination therapy for solid tumor indications.

## Methods

This research complies with ethical regulations and has been approved by the Dutch animal ethics committee (CCD) and the local Animal Welfare Body of the Leiden University Medical Center (LUMC) and Radboud University Medical Center (UMC).

### Study design

Two fully syngeneic subcutaneous solid mouse tumor models were used to study the effect of Fc-silenced CD3xTRP1 bsAb in combination with SLP vaccination. Anti-tumor efficacy was investigated in completely syngeneic mouse models and mice were sacrificed after humane endpoints (tumor volume >1000 mm^3^, ulcerated tumor, or a body conditioning score of BC2 or lower), or the pre-determined maximal study duration were reached. During our studies, tumor sizes did not exceed 2000 mm^3^, which is the maximal tumor size allowed by our ethical committee. To prevent the occurrence of unwanted immune responses and remain consistent throughout the study, we have only used male mice as the B16 tumor is derived from a male mouse. T-cell trafficking was analyzed using in vivo imaging, flow cytometry and IHC, after sacrificing the mice at pre-determined time points. Finally, in vivo imaging, high dimensional flow cytometry and Nanostring transcriptomics analysis was used to investigate the activation of T cells and other immune cells in the TME after sacrificing the mice at pre-determined time points. All mouse studies were performed with mice of the same sex and age. Sample sizes were chosen empirically to ensure adequate statistical power and were in line with the standards for the specific techniques. Treatments were randomized based on tumor volume if possible, or otherwise randomly distributed over the cages to minimize cage effects. Apart from multiple treatments being present in the same cage, no additional blinding was used. For ex vivo intratumoral analyses we excluded tumors <3 × 3 × 3 mm (length * width * height). The number of experimental replicates is indicated in the figure legends.

### Cell lines

The B16F10 murine melanoma cell line was purchased from the ATCC (CRL-6475). The KPC3 murine pancreatic carcinoma with mutant *K-ras* and *p53* is derived from a primary tumor in a C57BL/6 mouse^49^. The MC38 cell line is a chemically-induced murine colon carcinoma and was obtained from Prof. F. Ossendorp (LUMC, Leiden, the Netherlands)^50^. KPC3-TRP1 and MC38-TRP1 were obtained as previously described^10,51^ and were cell-sorted for TRP1 expression using an anti-TRP1 antibody (TA99). All cell lines were cultured as previously described^10^, authenticated by IDEXX bioanalytics using short tandem repeat markers and frequently tested for mycoplasma.

### Bispecific antibody generation

CD3 bsAbs were generated with controlled Fab-arm exchange as previously described, using the 145-2C11 clone recognizing CD3ε and the TA99 clone recognizing TRP1^10,52^. The CD3xTRP1 bsAb was generated as an IgG2a isotype with L234A-L235A (LALA) mutations to silence Fc-mediated effector functions^53,54^.

### Mice

8 Weeks old C57BL/6 mice were purchased from Charles River, the Netherlands. CXCR3 KO mice were purchased from Jackson Laboratories (stock number 005796). T-cell receptor (TCR) transgenic mice containing the chicken ovalbumine OVA_257-264_/H-2K^b^ specific receptors (designated as OT-1) were purchased from Jackson Laboratories (stock number 003831) and bred to express the congenic marker CD45.1. Albino C57BL/6 mice lacking the tyrosinase gene, Jackson stock number (000058), and TbiLuc mice (dual T cell luciferase transgenic mice) were generated at the Leiden University Medical Centre as previously described (internal reference number T1700014)^12^. F1 crosses of TbiLuc x OT-1 were used for experiments. All mice strains were bred at the Leiden University Medical Centre animal facility. All mouse experiments except the bsAb biodistribution study were performed at the animal facility of the LUMC, the Netherlands. The bsAb biodistribution study was performed at the animal facility of the Radboud UMC, the Netherlands. The health status of the animals was monitored over time and all animals were tested negative for agents listed in the FELASA (Federation of European Laboratory Animal Science Associations) guidelines for specific-pathogen free mouse colonies^55^. Mice were housed in the following conditions: dark/light cycle 06.30-07.00 sunrise, 07.00–18.00 day time, 18.00–18.30 sunset, 18.30–06.30 night time; 20–22 °C; 50–60% humidity. All mouse studies were approved by the Dutch animal ethics committee (CCD) and the local Animal Welfare Body of the LUMC or Radboud UMC on the permit numbers AVD116002015271 and AVD11600202010004, or AVD1030020209645, respectively. Experiments were performed in accordance with the Dutch Act on Animal Experimentation and EU Directive 2010/63/EU (“On the protection of animals used for scientific purposes”).

### Mouse treatments for survival and infiltration studies

For subcutaneous B16F10 and KPC3-TRP1 tumors, tumor cells (50,000 for survival experiments, 100,000 for TME studies) were injected s.c. in 100/200 µL PBS (Fresenius Kabi) with 0.1% BSA (Sigma) in the right flank. For MC38-TRP1 tumors, mice were injected s.c. with 500.000 MC38-TRP1 cells in PBS with 0.1% BSA in the right flank. For orthotopic KPC3-TRP1 tumors, mice received 0.1 mg/kg buprenorphine in PBS (Richter Pharma) s.c. prior to surgery and were then anesthetized with isoflurane. Next, a small incision was made and the top of the pancreas was taken out of the peritoneum using a cotton swab and injected with 10.000 KPC3-TRP1 tumor cells in PBS with 0.1% BSA. Finally, the incision was closed with sutures and the wound cleaned with PBS. With the exception of the orthotopic tumors, tumor sizes were measured three times a week by caliper and calculated by multiplying length × width × height. Mice were euthanized when the humane endpoint was reached.

Mice were treated with 12.5 µg CD3xTRP1 (approximately 0.5 mg/kg) i.p. in 100/200 µL PBS. For T-cell transfer, lymphocytes from spleens and lymph nodes of naïve TCR-transgenic OT-1 mice were isolated and enriched for T lymphocytes by nylon wool. 1 × 10^6^ Enriched T cells were transferred by i.v. injection into the tail vein in 200 µL PBS.

Peptides for immunizations were produced in house and were used with imiquimod and IL-2 as adjuvant, unless indicated otherwise. Before immunization, mice were anesthetized by an i.p. injection of a mixture of xylazine and ketamine in 100 µL PBS. Immunization with synthetic long peptides was performed on shaved left flanks by s.c. injections of 150 μg gp100_20–39_ peptide (AVGALKVPRNQDWLGVPRQL homologous human sequence), or chicken ovalbumine OVA_241–270_ peptide (“OVA”) (SMLVLLPDEVSGLEQLESIINFEKLTEWTS) dissolved in 100 µL PBS. As adjuvant, 60 mg of 5% imiquimod-containing cream Aldara (3 M Pharmaceuticals) was simultaneously topically applied on the skin at the injection site. Recombinant human IL-2 (600,000 IU, Proleukin®, Clinigen) was injected i.p. in 100 µL PBS at the day of the second immunization and one day later.

For the MC38-TRP1 survival experiment, mice were immunized with 100 μg OVA_241–270_ peptide or Rpl18_115–132_ (KAGGKILTFDRLALESPK) neoantigen peptide with 20 μg CpG (ODN-1826, Invivogen) in 50 μL PBS s.c. in the tailbase.

Immunization with LCMV-specific gp34 peptide was performed by injection of 100 μg peptide containing the underlined gp_33–41_ epitope VITGIKAVYNFATCGIFALIS with 20 μg CpG in 50 μL PBS s.c. in the tailbase. For viral infection, mice were injected with 2 × 10^5^ PFU LCMV-Armstrong virus i.p. in 100 μL PBS. The LCMV-Armstrong virus was propagated on BHK cells and viral titers were determined by plaque assays on Vero cells as described^56^.

For infection with influenza HKx31 virus, mice were anesthetized by inhalation of isoflurane (4%) and 100× median tissue culture infectious dose (TCID_50_) in 20 μL PBS was administered intranasally. HKx31 virus was kindly provided by Dr. Klaas van Gisbergen^21^. Boosting the immune response against HKx31 was performed by administration of 2,000,000 × TCID_50_ heat-inactivated (1 h at 70 °C) HKx31 in 200 μL PBS in the contralateral flank with imiquimod and IL-2 as adjuvant.

### In vivo bioluminescence imaging studies

Albino C57BL/6J mice were injected with 80,000 KPC3 tumor cells in the left flank and 80,000 KPC3-TRP1 cells in the right flank. OT-1xTbiLuc cells were injected i.v. in albino C57BL/6J mice as described above for OT-1 T cells. Immunization was performed by injection of 150 μg OVA_241-270_ peptide and 20 μg CpG in 50 μL PBS s.c. in the tailbase and bsAb treatment was administered by i.p. injection of 12.5 µg CD3xTRP1 in 100 μL PBS.

OT-1xTbiLuc T cells were visualized in vivo with an IVIS Spectrum small animal imager (PerkinElmer). These OT-1 T cells express two different types of luciferase: 1) under the control of the CD2 promoter, these T cells constitutively express a click-beetle luciferase (CBG99), which converts D-Luciferin (D-Luc), reflecting T-cell distribution, and 2) under the regulation of an NFAT promoter, they express an inducible firefly luciferase (PPyRE9), which converts CycLuc1, reflecting T-cell activation. Mice were injected with 4.88 mg/kg Cycluc1 (Aobious, Bio-Connect) in 100 µL PBS s.c. in the scruff of the neck and anesthetized via isoflurane inhalation (4% induction, 1.5% maintenance). Bioluminescence was measured 15 minutes after the injection using an open filter with an automatic exposure time. Then, mice were injected with 150 mg/kg D-Luciferin (Synchem) in 100 µL PBS s.c. in the scruff of the neck and while remaining under isoflurane-induced anesthesia. Bioluminescence was measured 15 min later using a 540 nm filter and an automatic exposure time. Signal quantification in specific regions of interest (ROIs) was performed by using fixed-size ROIs throughout the experiment using LivingImage 4.2 software (PerkinElmer).

### CD3xTRP1 biodistribution studies

CD3xTRP1 was conjugated with isothiocyanatobenzyl-diethylenetriaminepentaacetic acid (ITC-DTPA, Macrocyclics) and radiolabeled with indium-111 chloride (^111^InCl_3_, Curium) as described previously^57^. Analysis of radiolabeling efficiency using photosensitive plates (Fuji MS, Cytiva), a phosphor imager (Typhoon FLA 7000, GE) and AIDA v.4.21.033 software (Raytest, Straubenhardt, Germany) indicated an efficiency of 98.3%.

12.5 μg [^111^In]-DTPA-CD3xTRP1 (10-15 MBq) was injected i.p. and OT-1 cells and OVA vaccination were administered as described above. [^111^In]-DTPA-CD3xTRP1 in vivo distribution was visualized using SPECT/CT imaging (U-SPECT II/CT, MILabs) under 2% isoflurane inhalation anesthesia on a heated bed (38 °C). The following SPECT acquisition settings were used: 1.0 mm diameter pinhole mouse high sensitivity collimator (acquisition times 15 min for 6 h, 25 min for 24 h, 35 min for 72 h, and 45 min for 168 h time point), and CT acquisition settings: 160 µm spatial resolution, 615 µA, and 65 kV. The images were reconstructed with the MILabs software verson 2.04 applying energy windows at 171 keV (range 154–188 keV) and 245 keV (range 220–270 keV), 3 iterations, 16 subsets and a 0.2 mm voxel size. The Inveon Research Workplace software package (version 4.1) and a Gaussian 3 × 3 × 3 voxels filter were used for the SPECT image to create Maximum-intensity projections. To quantify the SPECT/CT data, a calibration curve was created by scanning a series of known activities using the same SPECT/CT settings.

To analyze the biodistribution of [^111^In]In-DTPA-CD3xTRP1 to various tissues, relevant tissues were harvested from the mice at the indicated time points. Subsequently, the tissue samples were weighed (XPE105DR, Mettler Toledo) and radioactivity concentrations were measured with a well-type gamma counter (Wallac 2480 wizard, PerkinElmer). Aliquots of injection fluid were measured as reference to calculate normalized uptake values as percentage injected dose per gram tissue.

### Flow cytometry

Tumors, spleen, or blood were harvested and immune cells were analyzed with flow cytometry. Single-cell suspensions from KPC3 and KPC3-TRP1 tumors were prepared by physical fragmentation followed by 10 min incubation with 2.5 mg/mL liberase ^TM^ (Roche) at 37 °C in a humidified atmosphere containing 5% CO_2_. Then, cell suspensions from KPC3 and KPC3-TRP1 tumors, or whole B16F10 tumors and spleens were minced through a 70 µm cell strainer (Falcon) and plated for FACS staining. Mouse Fc-receptors were blocked by Rat Anti-Mouse CD16/CD32 (Clone 2.4G2, BD) for 15 min at 4 °C. Viability was assessed with the Zombie UV^TM^ Fixable Viability Kit (Biolegend) or the LIVE/DEAD™ Fixable Aqua Dead Cell Stain Kit in PBS before surface staining. APC-conjugated H-2K^b^ OVA_257-264_ SIINFEKL, H-2K^b^ gp_34-41_ AVYNFATC, H-2K^b^ SARS-CoV-2 Spike_539-546_ VNFNFNGL, or H-2D^b^ PA_224-233_ SSLENFRAYV, or PE-conjugated H-2D^b^ gp_33-41_ KAVYNFATC were stained for 30 min at RT in PBS supplemented with 0.5% BSA + 0.002% sodium azide (FACS buffer). Then, other surface markers were stained in FACS buffer for 20 min at 4 °C. Next, cells were fixed and permeabilized for intracellular marker staining using the FoxP3/Transcription Factor Staining Buffer Set (eBioscience) according to manufacturer’s protocol. Finally, cells were resuspended in FACS buffer and measured on a Fortessa cytometer (BD bioscience), or Aurora 5L spectral flow cytometer (Cytek) and analyzed with FlowJo software v10.8.1 (Treestar) or OMIQ software, respectively. An overview of all the antibodies used for flow cytometry is shown in Supplementary Table 1.

### Immunohistochemistry

Tumors were isolated from mice, cut into slices with razor blades and directly fixed in formalin, followed by embedding in paraffin. Tumor tissues were sliced into 4 µm sections and mounted on adhesive slides (VWR Chemicals). Sections were then deparaffinized and rehydrated after which endogenous peroxidase activity was blocked with 0.3% hydrogen peroxidase solution (Merck Millipore) in methanol (VWR Chemicals) for 20 min. Antigen retrieval was performed in 0.01 M Sodium Citrate solution (Merck Millipore, pH 6.0) in the microwave for 10 min. Non-specific binding was blocked using SuperBlock™ (PBS) Blocking Buffer (ThermoFisher) at RT for 30 min. Tumor slides were incubated with Rat anti-Mouse CD8 (clone 4SM15, ThermoFisher) antibody diluted in 1% BSA in PBS at 4 °C overnight. Slides were then washed (0.05% Tween in PBS) and incubated with biotinylated Rabbit anti-Rat IgG (Abcam) at room temperature for 1 h. The biotinylated antibody was labeled using the VECTASTAIN® Elite ABC-HRP Kit (Vectorlabs) according to manufacturer’s protocol. Nuclear counterstaining was performed with filtered Mayer’s Haematoxylin staining (ThermoFisher) at room temperature for 10–15 s. Antibody binding was detected with the Liquid DAB+ substrate chromogen system (DAKO, Agilent) and DAB staining was quantified using ImageJ software. For characterization of the depth of intratumoral CD8^+^ T-cell infiltration, IHC scans were analyzed in HALO software (Indica Labs). CD8 IHC image scans were annotated to define the tumor region and were digitally segmented into ten circular layers with identical width. By using the predesigned CytoNuclear v2.0.9 algorithm, CD8^+^ T-cell densities were obtained for all ten circular layers separately. Data were plotted as CD8^+^ cells/mm^2^ tissue per circular layer.

### Nanostring transcriptomic analysis

RNA was isolated from single-cell suspensions of tumors using a NucleoSpin RNA kit (Macherey-Nagel) according to manufacturer’s protocol. The quality and yield after sample preparation were determined using the Agilent DNF-474 HS NGS Fragment Kit and measured with the Fragment Analyzer. The size of the resulting products was consistent with 95% of the fragments >300 bp. We loaded 300 ng RNA on nCounter PanCancer Immune Profiling cartridges (catalog # 115000142, Nanostring) and measured the fluorescent barcodes on a nCounter Analysis System (NanoString) according to the manufacturer’s instructions. Data were processed and normalized using nSolver Analysis Software (V4.0) and the Advanced Analysis module (V2.0, NanoString). NanoString-defined markers were used to analyze scores for cell types and immune pathways. Benjamini-Hochberg adjusted p values were used to decrease the false-discovery rate.

### Statistical analysis

Data are presented as mean ± SEM as stated in the figure legends. Statistical significance was determined as indicated in the figure legends using GraphPad Prism 9 software or for the Nanostring transcriptomic analyses using nSolver V4.0 software, with *P* < 0.05 being considered as statistically significant, *P < 0.05, **P < 0.01, ***P < 0.001 and ****P < 0.0001.

### Reporting summary

Further information on research design is available in the Nature Portfolio Reporting Summary linked to this article.

### Supplementary information

Supplementary Information

Peer Review File

Description of Additional Supplementary Files

Supplementary Data 1

Supplementary Data 2

Supplementary Data 3

Supplementary Data 4

Reporting Summary

### Source data

Source Data

## Data Availability

All data are available within the Article, Supplementary Information or Source Data file. Source data are provided with this paper.
