# Supplementary material for: T-cell stimulating vaccines empower CD3 bispecific antibody therapy in solid tumors
Source: Nat Commun. 2024 Jan 2;15:48. doi: 10.1038/s41467-023-44308-6 (PMC10761684; doi:10.1038/s41467-023-44308-6)
Supplement: Supplementary file 1 — Supplementary Information [file 41467_2023_44308_MOESM1_ESM.pdf]

Supplementary Figure 1 (related to Figure 1)

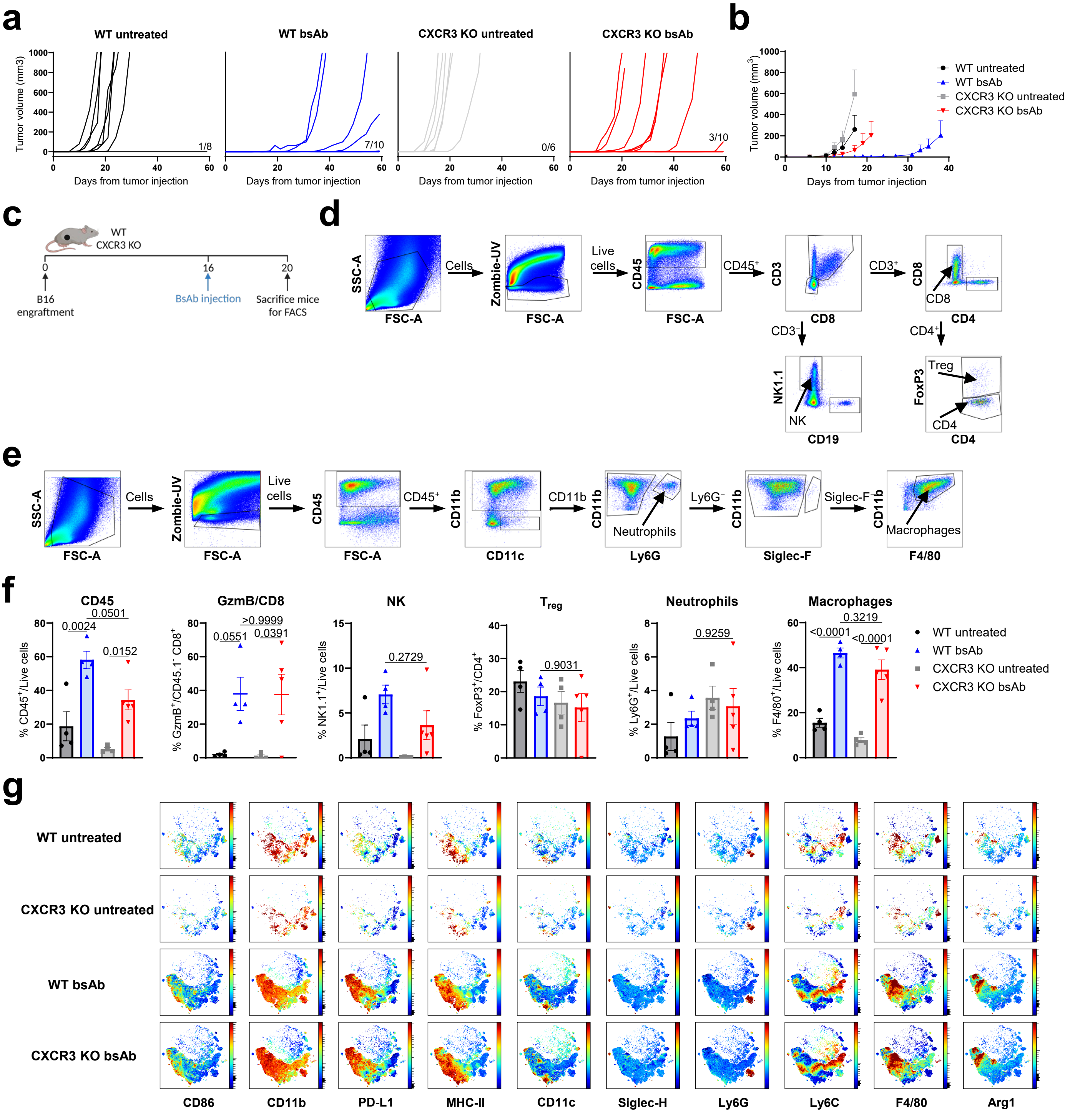

Supplementary Figure 1. Tumor growth curves, flow cytometry gating and results of infiltration data in WT and CXCR3 KO mice. (**a-b**) Tumor growth curves for indicated groups belonging to figure 1b depicted as individual growth curves (a), or mean tumor volumes (b). (**c**) Treatment schedule for TME analysis of WT and CXCR3 KO mice bearing B16F10 tumors receiving CD3xTRP1 treatment by using flow cytometry. (**d**) Gating strategy for lymphoid cells used for figures 1c, 5b-c and f-g, 6c-d and supplementary figures 1f, 6a and d, 7a and h, 8c-d, 9a and g and 10c-d. (**e**) Gating strategy for myeloid cells used for supplementary figure 1f. (**f**) Frequencies of intratumoral immune cells for the indicated groups bearing B16F10 tumors treated with CD3xTRP1 on day 16 and sacrificed on day 20. (**g**) Expression of indicated markers (mean fluorescence intensity) from live $CD45^+$ cells for indicated groups in opt-SNE plots, generated using OMIQ software. Color scale indicates expression: red, high; blue, low. Data represented as mean ± SEM (b and f). Panel b (WT untreated (n=8), WT bsAb (n=10), CXCR3 KO untreated (n=6), CXCR3 KO bsAb (n=10)), panel f (WT untreated (n=4), WT bsAb (n=4), CXCR3 KO untreated (n=4), CXCR3 KO bsAb (n=5)). Significance was calculated with one-way ANOVA and Tukey's post-hoc tests comparing all groups (f). Source data are provided as a Source Data file.

Supplementary Figure 2 (related to Figure 1)

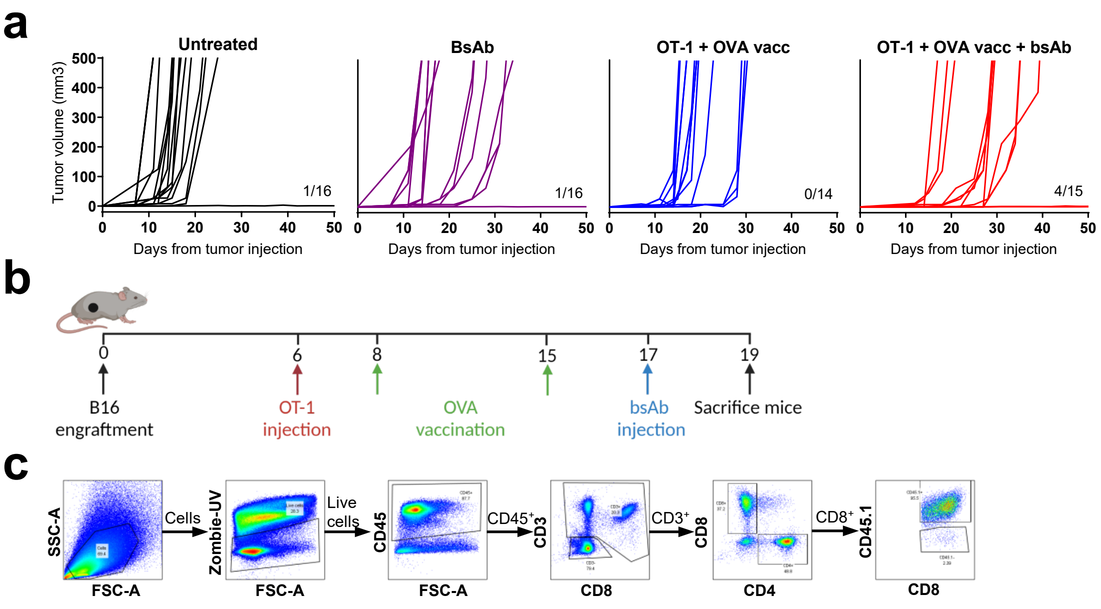

Supplementary Figure 2. Tumor growth curves, treatment schedule and flow cytometry gating for OT-1, OVA peptide vaccination and CD3xTRP1 treatment. (**a**) Individual tumor growth curves for indicated groups belonging to figure 1e. (**b**) Treatment schedule for mice bearing B16F10 tumors receiving OT-1, OVA peptide vaccination and/or CD3xTRP1 treatment for subsequent TME analysis. (**c**) Gating strategy for lymphoid cells used for figures 1f, 3b, 4a-c and e and supplementary figures 3d, 4a and c. Source data are provided as a Source Data file.

Supplementary Figure 3 (related to Figure 2)

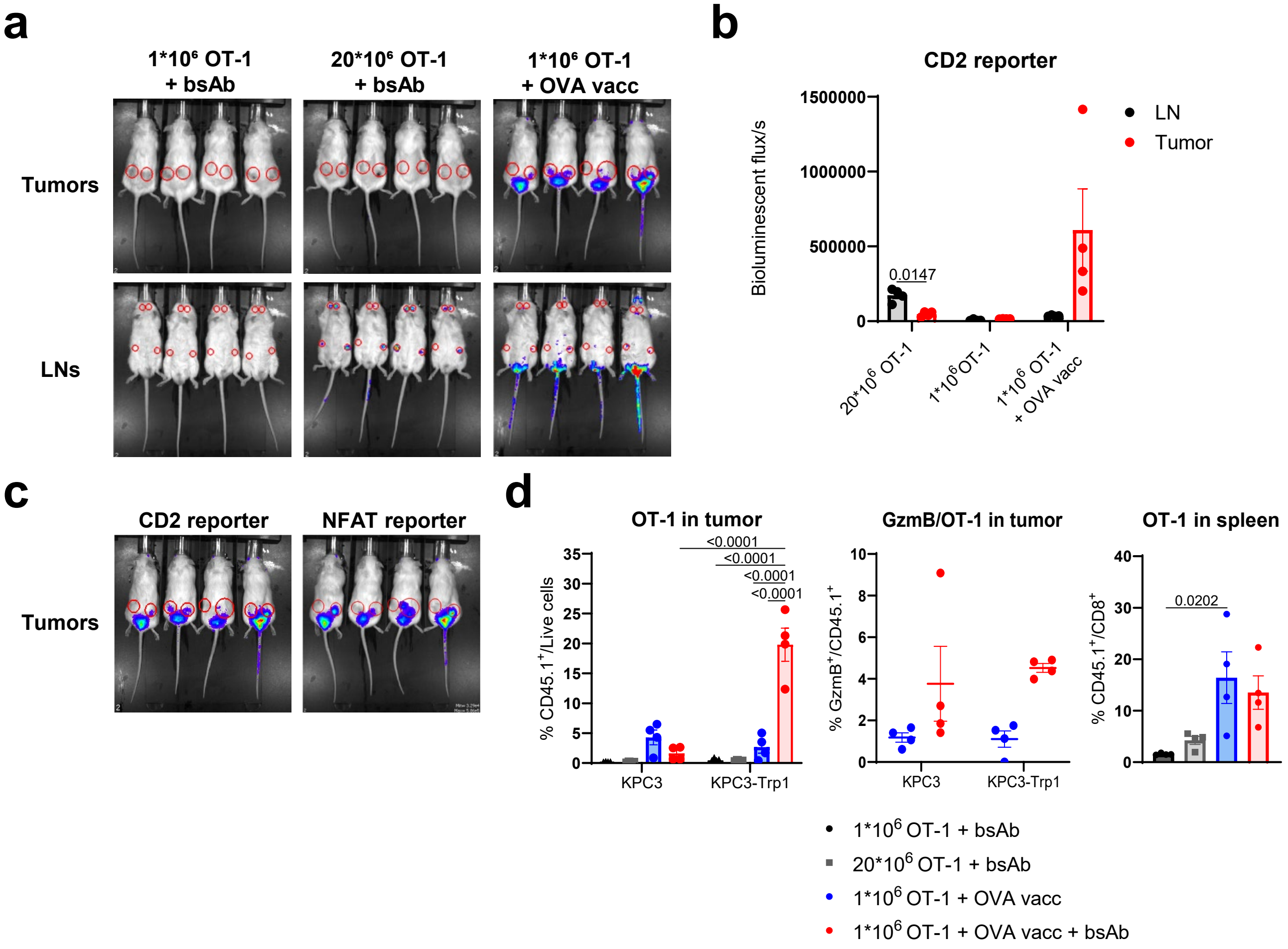

Supplementary Figure 3. Vaccine-activated non-specific T cells infiltrate the tumor, whereas naïve T cells home towards lymph organs (**a**) Representative IVIS images at day 19 according to the schedule in figure 2a. Red circles indicate tumors (left flank, KPC3; right flank, KPC3-TRP1) or LNs. Color gradient indicates expression level: blue, low; red, high. Signal at the tail base indicates vaccination site. (**b**) Quantification of bioluminescent flux from the CD2 reporter in lymph nodes and tumors on day 15 prior to CD3xTRP1 administration. Bar graphs represent the sum of the flux of the inguinal lymph nodes, or KPC3 and KPC3-TRP1 tumors, as indicated in the red circles in supplementary figure 3a. (**c**) Representative IVIS image from day 19 of mice that received OT-1 transfer, OVA vaccination and CD3xTRP1. Red circles indicate tumors: left, KPC3; right, KPC3-TRP1. Signals at tailbase of the mice likely reflect OT-1 accumulation around OVA antigen depots at the vaccination site. Color gradient indicates expression level: blue, low; red, high. (**d**) Frequencies of OT-1 T cells in tumor and spleen and fraction of GzmB positive OT-1 cells treated according to scheme in figure 2a. Data represented as mean ± SEM of n=4 (b and d). Significance was calculated using paired two-sided t-tests (b), or one-way ANOVA with Tukey's post-hoc tests comparing all groups (d). Source data are provided as a Source Data file.

Supplementary Figure 4 (related to Figure 3)

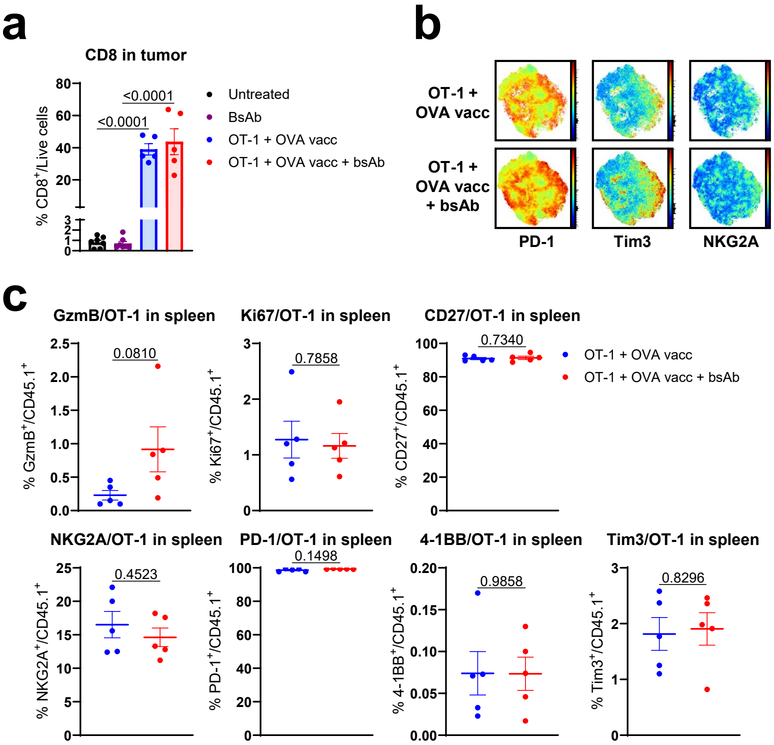

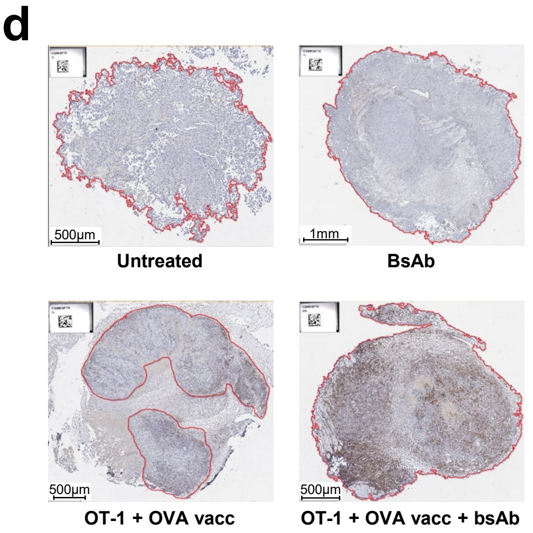

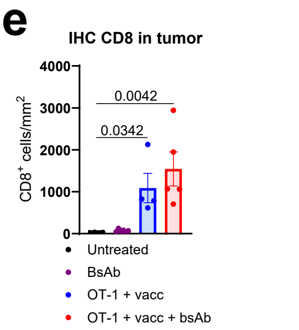

f

OT-1 + OVA + bsAb vs OT-1 + OVA

	Log2 FC	Adj. p
Cxcl9	4.88	1.21E-03
Ifng	4.32	5.60E-04
Cxcl10	3.72	3.65E-04
Irf4	3.49	1.51E-04
Cxcl11	3.41	6.41E-04
Prf1	3.35	6.56E-05
Pdcd1	3.35	2.59E-04
Ctla4	3.34	1.51E-04
Lag3	3.27	6.56E-05
Havcr2	3.25	2.57E-04
Gzma	3.25	1.70E-03
Gzmb	3.18	6.86E-04
Tigit	2.85	3.34E-04
Cd3g	2.85	5.57E-04
Gzmk	2.71	1.92E-03
Cd69	2.7	7.17E-04
Cd8a	2.66	3.08E-04

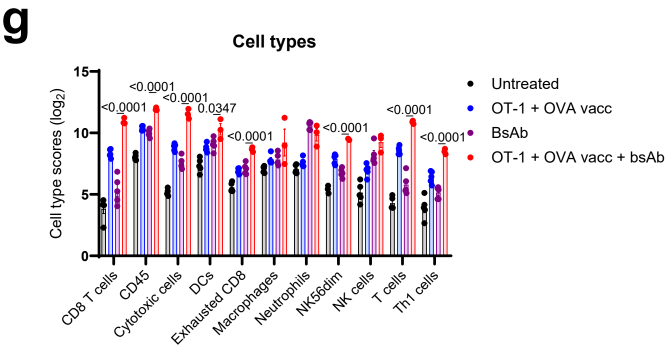

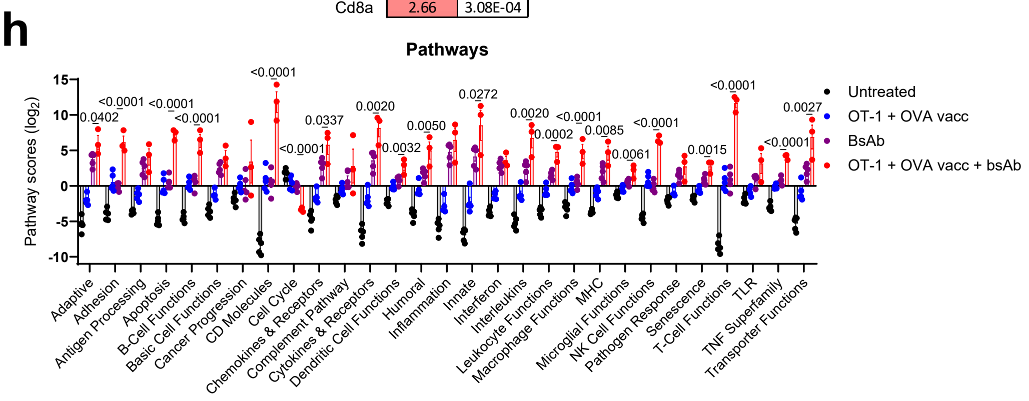

Supplementary Figure 4. Vaccine-activated OT-1 T cells are locally activated in the tumor after CD3xTRP1 administration. (**a**) Frequency of total tumor infiltrating CD8 T cells in KPC3-TRP1 tumors according to the treatment schedule in figure 3a. (**b**) Expression of T-cell activation markers (mean fluorescence intensity) in opt-SNE plots, generated using OMIQ software, from pre-gated OT-1 T cells in B16F10 tumors from mice that received OT-1 transfer on day 6, OVA vaccination on day 8 and 15, CD3xTRP1 on day 17 and were sacrificed on day 19. OT-1 cells were pre-gated based on CD45.1 expression and then further selected on selective abundance in the tumor to avoid mixing up systemic and local effects. Color scale indicates expression: red, high; blue, low. (**c**) Expression of T-cell activation markers on OT-1 T cells in the spleen from mice bearing KPC3-TRP1 tumors. (**d and e**) Representative images of tissue slide staining for CD8 of B16F10 tumors (according to treatment schedule described in supplementary figure 2b) (d) and quantification (e). Red line marks tumor region used for quantification. (**f**) Nanostring transcriptomics analysis with the PanCancer Immune Profiling Panel, showing a selected list of differentially expressed genes. (**g and h**) Representation of differential gene expression in KPC3-TRP1 tumors from Nanostring analysis in scores for cell types (f) and immune pathways (g). Statistical significance in (f and g) is only shown for the comparison between bsAb and OT-1 + OVA vacc + bsAb. Data represented as mean ± SEM (a, c, e, g and h). Panel a and c (Untreated (n=7), BsAb (n=7), OT-1 + OVA vacc (n=5), OT-1 + OVA vacc + bsAb (n=5)), panel e (Untreated (n=4), BsAb (n=6), OT-1 + OVA vacc (n=4), OT-1 + OVA vacc + bsAb (n=5)), panel f-h (Untreated (n=5), BsAb (n=5), OT-1 + OVA vacc (n=5), OT-1 + OVA vacc + bsAb (n=3)). Significance was calculated using one-way ANOVA with Tukey's post-hoc tests comparing all groups (a, e, g and h), or unpaired two-sided t-tests either without (c) or with Benjamini-Hochberg correction for multiple comparisons (f). Source data are provided as a Source Data file.

Supplementary Figure 5 (related to Figure 3)

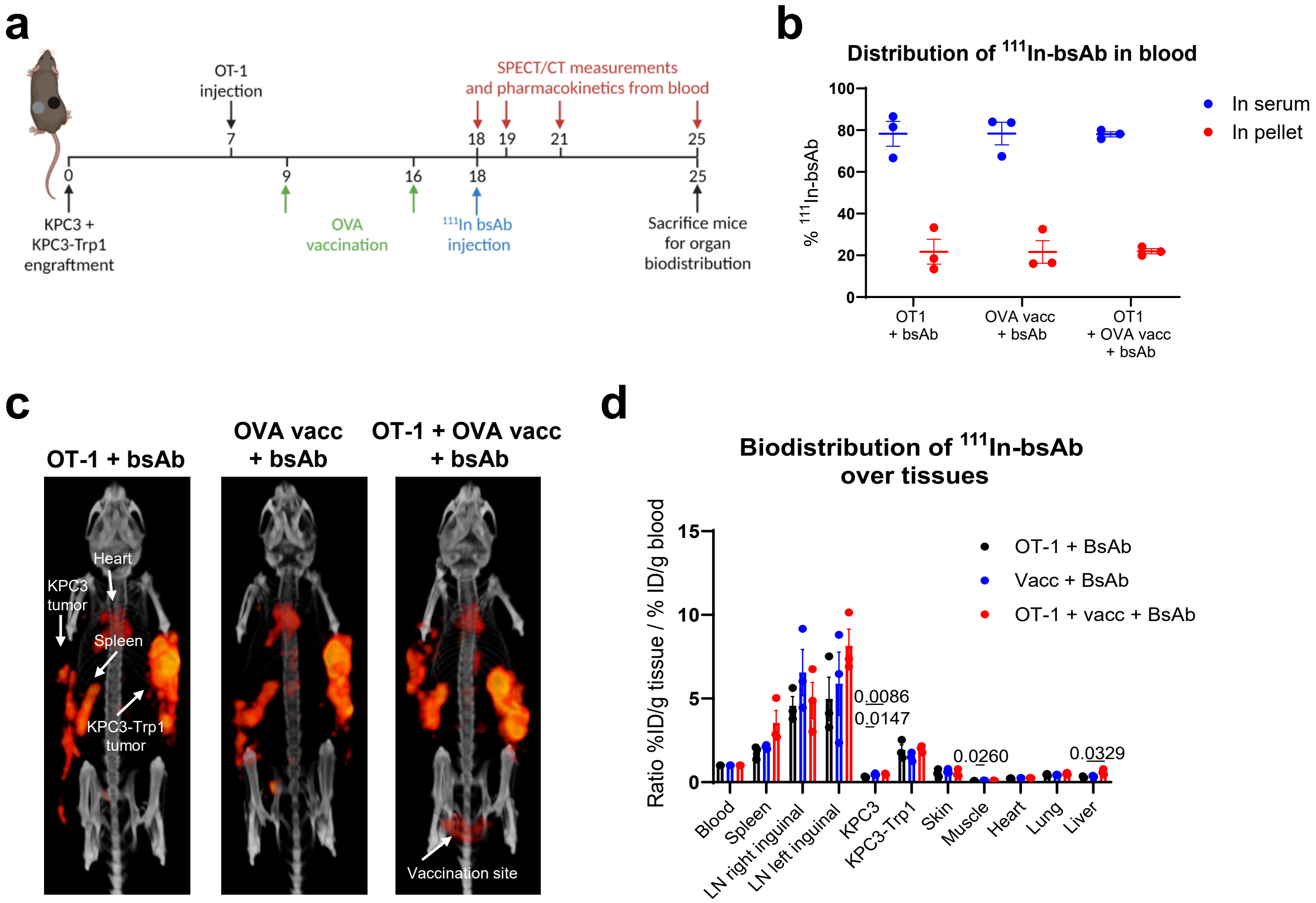

Supplementary Figure 5. Biodistribution of radiolabeled CD3xTRP1 in combination with OT-1 T cells and OVA peptide vaccination. (**a**) Treatment scheme for mice bearing KPC3 and KPC3-TRP1 tumors on distinct flanks receiving treatment with OT-1 T cells, OVA peptide vaccination and radiolabeled CD3xTRP1. SPECT/CT imaging was performed at indicated time points. (**b**) Distribution of radiolabeled CD3xTRP1 in blood, divided over serum fraction and cell pellet, 24 hours after injection of CD3 bsAb. (**c**) Representative SPECT/CT images 24 hours after injection with radiolabeled CD3xTRP1 from dorsal perspective. Orange indicates radiolabeled CD3 bsAb, white arrows indicate most important tissues/locations. (**d**) Biodistribution of radiolabeled CD3xTRP1 over indicated tissues after normalization to blood. Data represented as mean ± SEM of n=3 (b and d). Significance was calculated using one-way ANOVA and Tukey's post-hoc tests comparing all groups (d). Source data are provided as a Source Data file.

Supplementary Figure 6 (related to Figure 4)

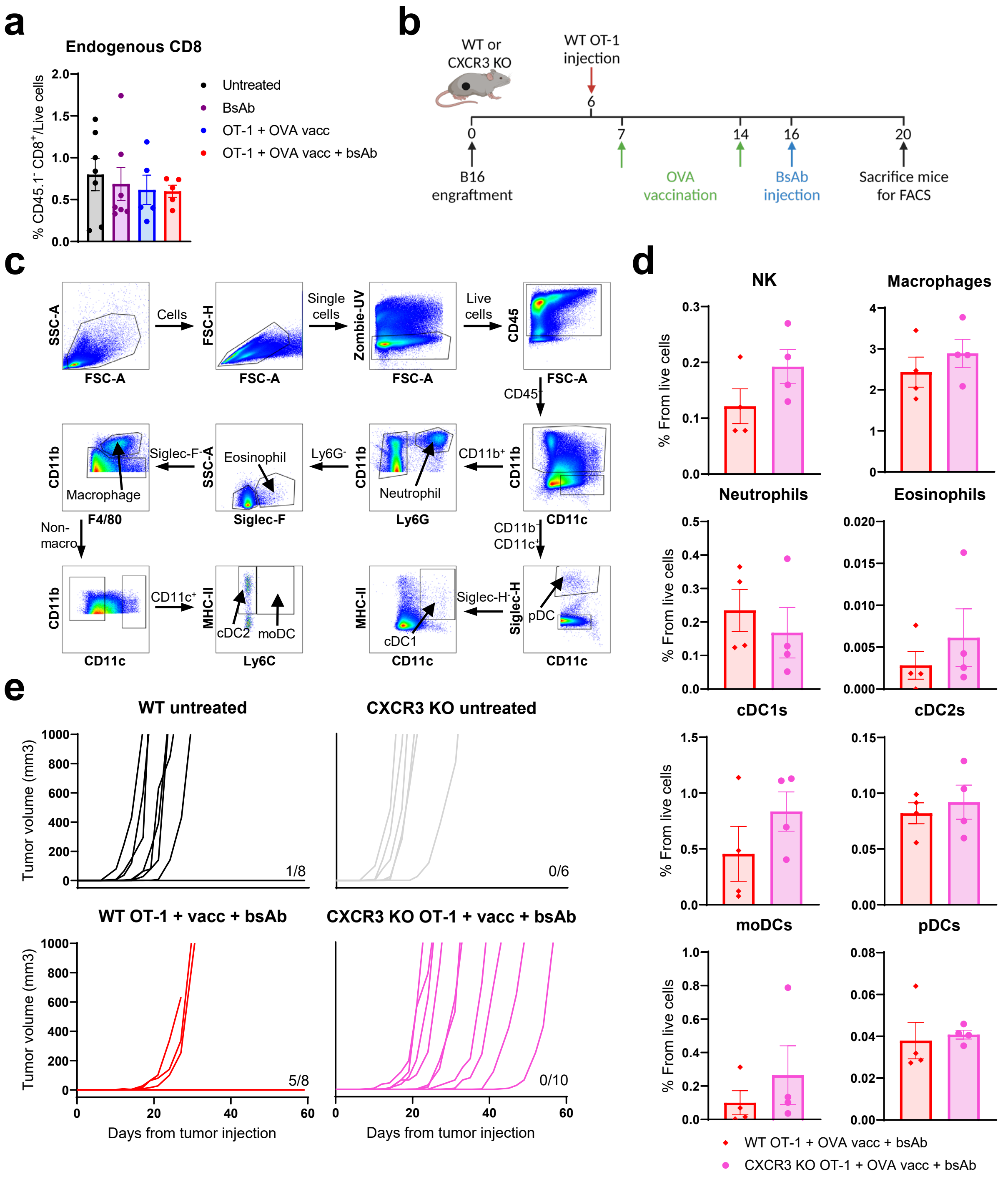

Supplementary Figure 6. Tumor growth curves, myeloid gating strategy and influx of immune cells for CXCR3 KO related experiments. (**a**) Frequency of endogenous CD8 T cells in KPC3-TRP1 tumors for indicated groups treated with OT-1 cell transfer, OVA vaccination and CD3xTRP1 according to the treatment schedule in figure 3a. (**b**) Treatment schedule for WT or CXCR3 KO mice treated with OT-1 T cells, OVA vaccination and CD3xTRP1 for TME analysis. (**c**) Gating strategy for myeloid cells used for figure 6e-f and supplementary figures 6d and 8e-h. (**d**) Frequencies of intratumoral immune cell populations within B16F10 tumors of WT or CXCR3 KO mice according to the treatment schedule in supplementary figure 6b. (**e**) Individual tumor growth curves for indicated groups belonging to figure 4f. Data represented as mean ± SEM (a and d). Panel a (Untreated (n=7), BsAb (n=7), OT-1 + OVA vacc (n=5), OT-1 + OVA vacc + bsAb (n=5)), panel d (n=4). Significance was calculated using one-way ANOVA followed by Tukey's post-hoc tests comparing all groups (a) and two-sided t-tests (d). Source data are provided as a Source Data file.

Supplementary Figure 7 (related to Figure 5)

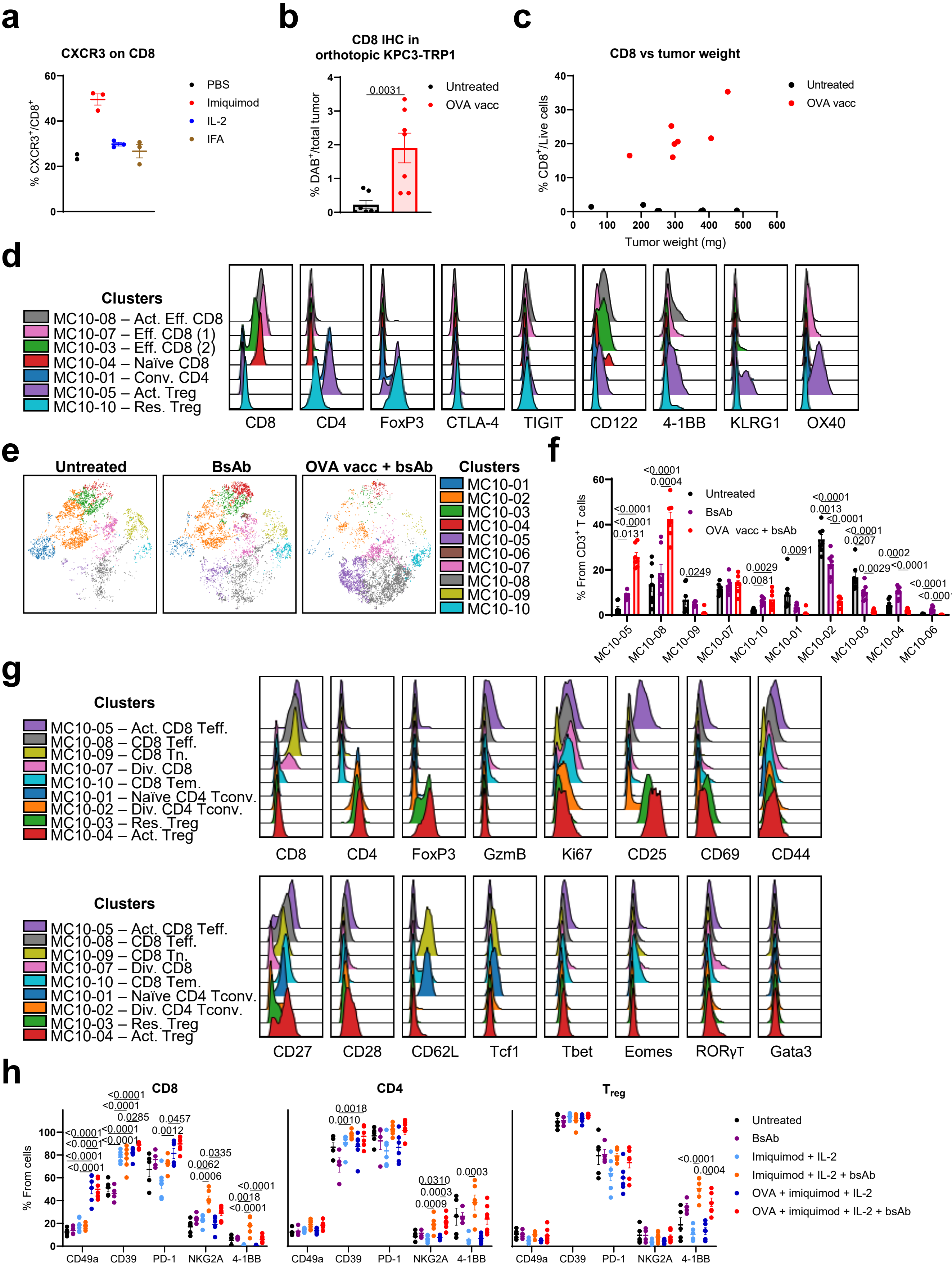

Supplementary Figure 7. Infiltration and activation of T-cell subsets in blood, KPC3-TRP1 and B16F10 tumors. (**a**) CXCR3 expression on CD8 T cells in blood on day 13 following administration of indicated adjuvants on day 0 and 7. IFA, incomplete Freund's adjuvant. (**b and c**) CD8 T-cell infiltrate in mice bearing orthotopic KPC3-TRP1 tumors in the pancreas, treated with OVA vaccination on day 9 and 16 and sacrificed on day 20, showing CD8 quantification of IHC staining shown in figure 5d (b), or correlation between CD8 infiltration shown in figure 5e and tumor weight (c). (**d**) Remainder of phenotypical marker expression for all metaclusters from figure 5h-j with >5% representation for any of the treatment groups (5j). Act., activated; Eff., effector; Conv., conventional; Res., resident. (**e-g**) Unsupervised clustering of pregated $CD3^+$ immune cells in KPC3-TRP1 tumors using OMIQ software (different flow cytometry panel than in figure 5h-j and therefore also different metaclusters and concordant annotation). Overlay of metaclusters on the opt-SNE results, showing 1000 $CD3^+$ cells per sample for a total of 7 samples per treatment group (e). Distribution of the metaclusters over the treatment groups (f). Selection of phenotypical marker expression for all metaclusters with >5% representation for any of the treatment groups (g). Act., activated; Eff., effector; Div., dividing; Conv., conventional; Res., resident. (**h**) Expression of phenotypical activation markers on T-cell subsets in B16F10 tumors. All mice were treated according to the treatment schedule in figure 5a. Data represented as mean ± SEM (a-c, f and h). Panel a (PBS (n=2), Imiquimod (n=3), IL-2 (n=3), IFA (n=3)), panel b, c and f (n=7), panel h (Untreated (n=5), BsAb (n=4), Imiquimod + IL-2 (n=6), Imiquimod + IL-2 + bsAb (n=6), OVA + imiquimod + IL-2 (n=6), OVA + imiquimod + IL-2 + bsAb (n=7)). Significance was calculated using unpaired two-sided t-tests (b), or one-way ANOVA and Tukey's post-hoc tests comparing all groups (f and h). Source data are provided as a Source Data file.

Supplementary Figure 8 (related to Figure 6)

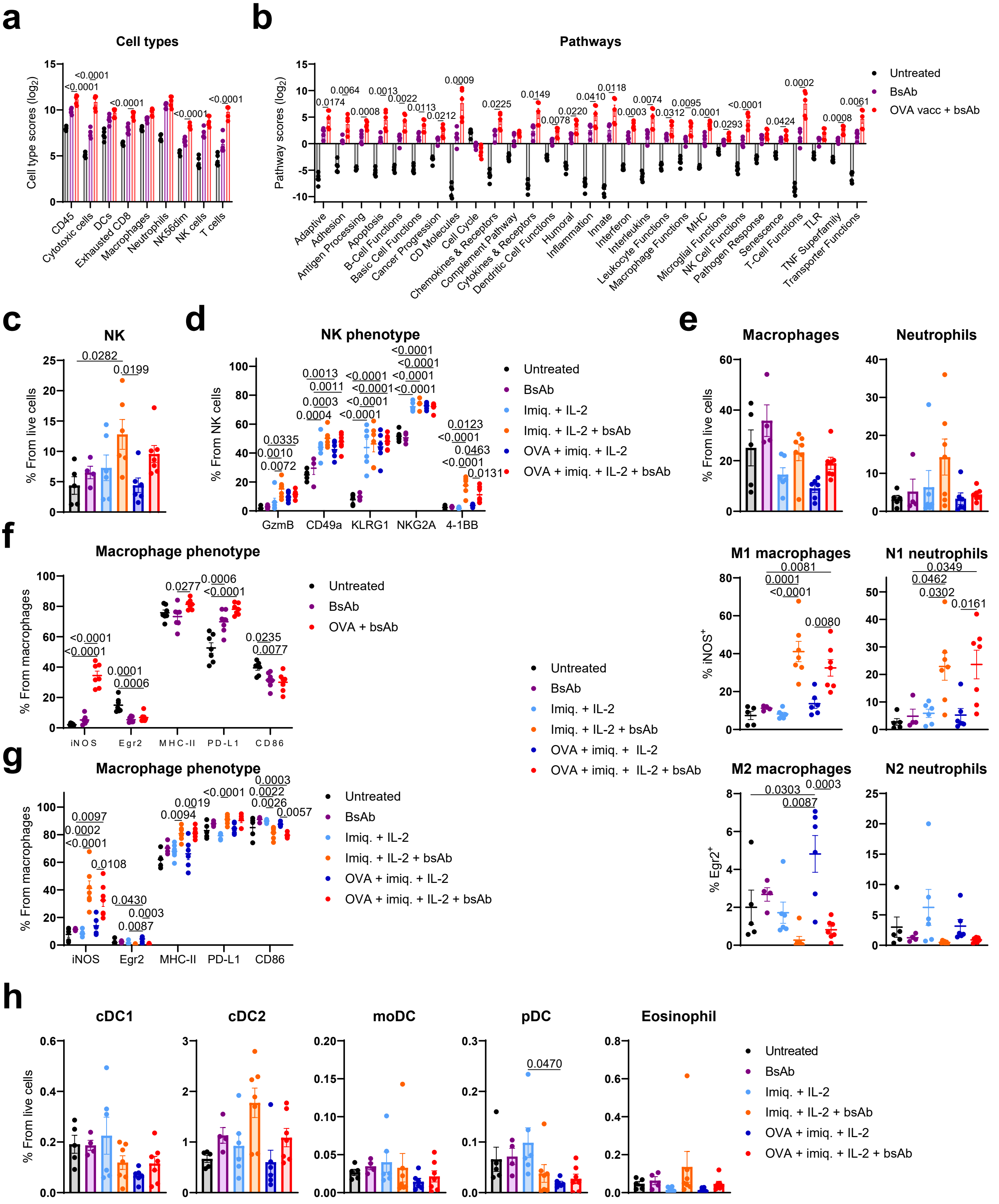

Supplementary Figure 8. Infiltration and activation of the innate immune cell subsets in OVA vaccination and CD3xTRP1-treated tumors. (**a and b**) Representation of differential gene expression from Nanostring analysis in scores for cell types (a) and immune pathways (b), using nSolver4.0 analysis software. Statistical significance in (a and b) is only shown for the comparison between bsAb and OVA vacc + bsAb. (**c and d**) Frequency (c) and phenotype (d) of intratumoral NK cells in B16F10 tumors. (**e-h**) Frequency and phenotype of myeloid immune cell subsets. Macrophage and neutrophil subsets in B16F10 tumors (e). M1 macrophages and N1 neutrophils were defined as inducible nitric oxide synthase (iNOS)-positive, M2 macrophages and N2 neutrophils were defined as early growth factor receptor 2 (Egr2)-positive. Phenotype of macrophages in KPC3-TRP1 tumors (f) and B16F10 tumors (g). Frequency of other myeloid immune cell subsets in B16F10 tumors (h). Flow cytometry markers and gating strategy are displayed in supplementary figures 1d and 6b. All mice (a-h) were treated according to the treatment schedule depicted in figure 5a. Imiq. indicates imiquimod; OVA represents OVA peptide. Data represented as mean ± SEM (a-h). Panel a and b (n=5), panel c-e and g-h (Untreated (n=5), BsAb (n=4), Imiq. + IL-2 (n=6), Imiq. + IL-2 + bsAb (n=6), OVA + imiq. + IL-2 (n=6), OVA + imiq. + IL-2 + bsAb (n=7)), panel f (n=7). Significance was calculated using one-way ANOVA and Tukey's post-hoc tests comparing all groups (a-h). Source data are provided as a Source Data file.

Supplementary Figure 9 (related to Figure 7)

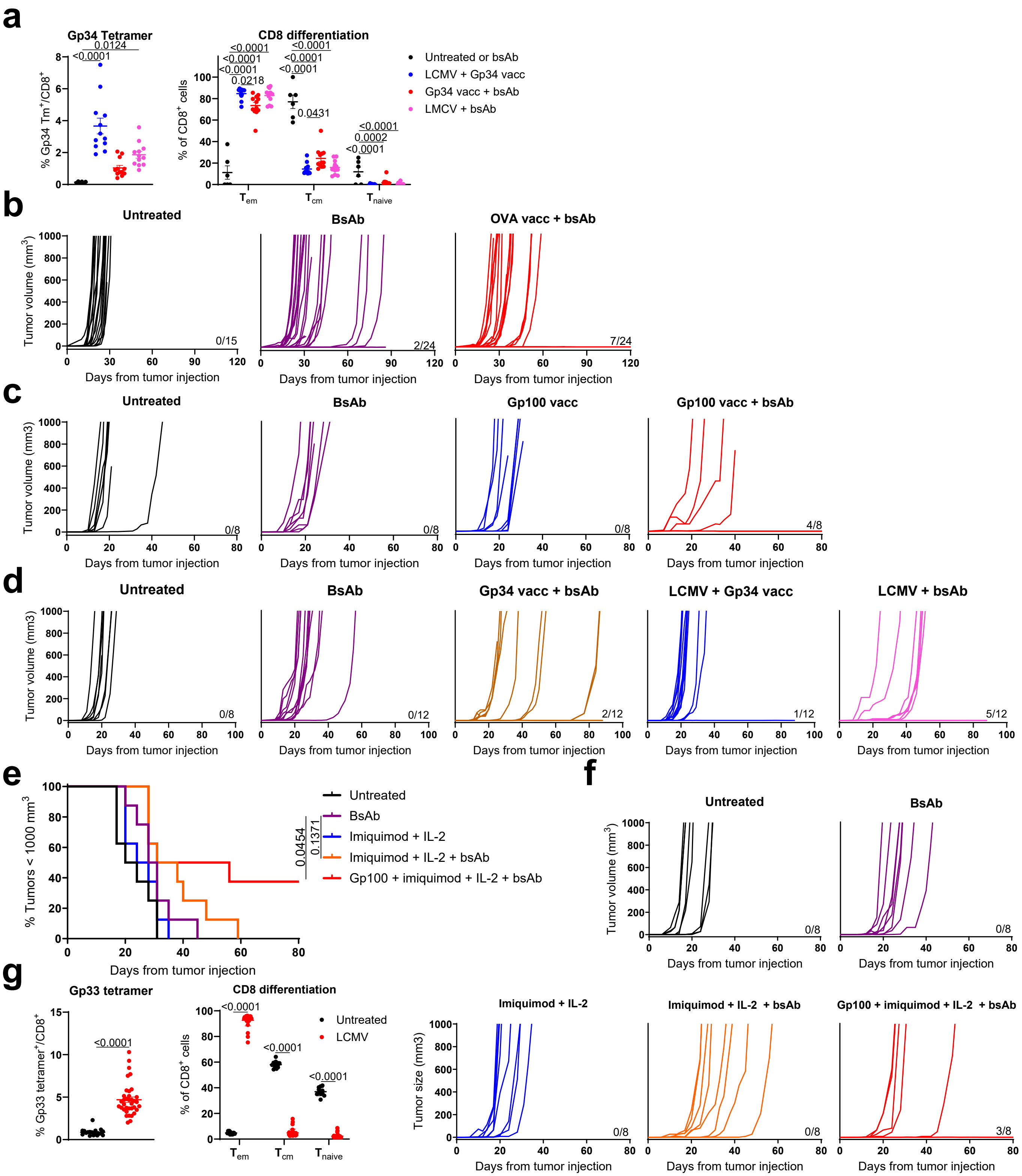

Supplementary Figure 9. Individual tumor growth curves for combination of OVA or Gp100 SLP vaccination and CD3xTRP1. (a, g) Treatment schedule according to figure 7c. (**a**) CD8 T cells in blood, showing Gp34 tetramer responses and differentiation of CD8 T cells on day 19. (**b-d**) Individual tumor growth curves for indicated groups belonging to figure 7a (b), figure 7b (c), or figure 7d (d). (**e-f**) Kaplan-Meier (e) and individual tumor growth curves (f) for mice treated with combination of adjuvant only or Gp100 vaccination with CD3xTRP1. Numbers indicate surviving mice at the end of the experiment. (**g**) CD8 T cells in blood, showing Gp33 tetramer responses and differentiation of CD8 T cells on day -22. Data represented as mean ± SEM (a and g). Panel a (Untreated or bsAb (n=6), LCMV + Gp34 vacc (n=12), Gp34 vacc + bsAb (n=12), LCMV + bsAb (n=12)), panel g (Untreated (n=18), LCMV (n=36)). Significance was calculated using one-way ANOVA and Tukey's post-hoc tests comparing all treatment groups (a), Mantel-Cox Log-Rank tests (e), or unpaired two-sided t-tests (g). Source data are provided as a Source Data file.

Supplementary Figure 10 (related to Figure 7)

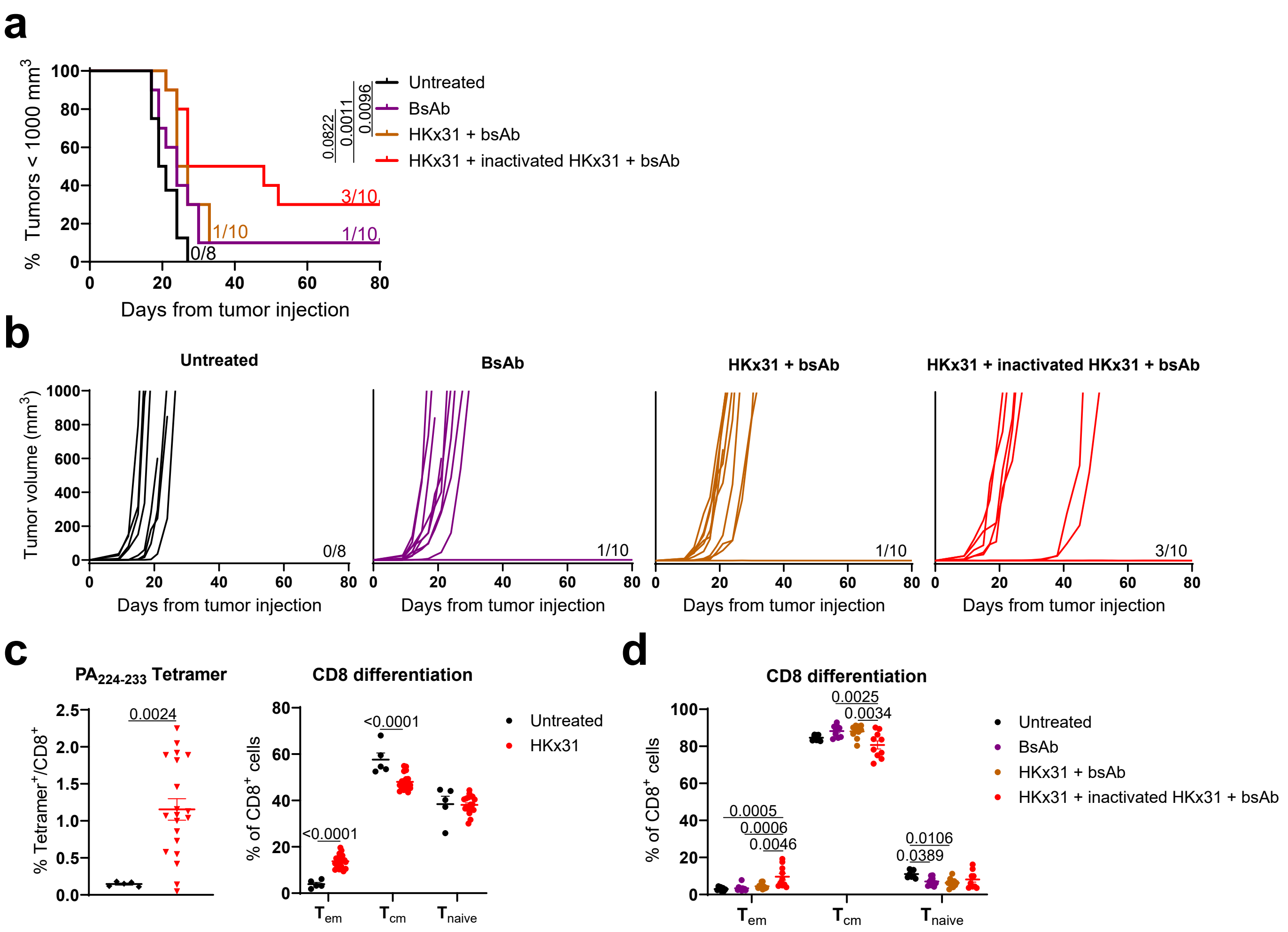

Supplementary Figure 10. CD8 T-cell responses to HKx31 infection and boost and tumor growth. (**a-d**) Treatment schedule according to figure 7c. (**a and b**) Kaplan-Meier (a) and individual tumor growth curves (b) for indicated groups. Numbers indicate surviving mice at the end of the experiment. (**c**) CD8 T cells in blood, showing HKx31-specific tetramer responses and differentiation of CD8 T cells on day -14. (**d**) Differentiation of CD8 T cells in blood on day 16 after mice received the HKx31 booster vaccine. Data represented as mean ± SEM (c and d). Panel c (Untreated (n=5), HKx31 (n=20)), panel d (Untreated (n=8), BsAb (n=10), HKx31 + bsAb (n=10), HKx31 + inactivated HKx31 + bsAb (n=10)). Significance was calculated with a Mantel-Cox Log-Rank tests (a), unpaired two-sided t-tests (c), or one-way ANOVA and Tukey's post-hoc tests comparing all treatment groups (d). Source data are provided as a Source Data file.

Supplementary Table 1. Overview of all used antibodies for flow cytometry experiments.

Marker	Fluorochrome	Clone	Catalog number	Lot number	Supplier	Fold dilution
4-1BB	APC	17B5	106110	B315095	Biolegend	200
Arginase 1	PE-Cy7	A1exF5	25-3697-82	2250751	Invitrogen	200
CD3	FITC	145-2C11	11-0031-85	4346374	Invitrogen	800
CD3	PE-Cy5	145-2C11	100310	B311089	Biolegend	500
CD4	BUV496	RM4-4	741051	0345594	BD	600
CD8	BUV395	53-6.7	563786	1207296	BD	400
CD8	BV711	53-6.7	100759	B332209	Biolegend	800
CD11b	BUV563	M1/70	741242	0251102	BD	1200
CD11c	BV605	HL3	563057	1084985	BD	200
CD19	Spark Blue	6D5	115566	B332505	Biolegend	800
CD25	BV421	PC61	102033	B284919	Biolegend	200
CD27	BV785	LG.3A10	124241	B321647	Biolegend	200
CD28	PE-Cy7	37.51	102126	B298200	Biolegend	200
CD39	PE	Duha59	143803	B291343	Biolegend	200
CD44	BV510	IM-7	103043	B333218	Biolegend	600
CD44	BV785	IM-7	103059	B346798	Biolegend	100
CD45	AF700	30-F11	103128	B327672	Biolegend	400
CD45.1	APC	A20	110714	B254042	Biolegend	400
CD49a	BUV737	Ha31/8	741776	1162852	BD	200
CD62L	BUV805	MEL-14	741924	1032098	BD	800
CD62L	BV421	MEL-14	104436	B356899	Biolegend	100
CD69	BUV737	H1.2F3	612793	1159821	BD	200
CD86	BUV496	PO3	750437	0353290	BD	200
CD115	PE/dazzle	AFS98	135528	B301555	Biolegend	100
CD122	PE-Cy5	TM-β1	123220	B294548	Biolegend	300
CTLA-4	BV421	UC10-4B9	106311	B378406	Biolegend	200
CXCR3	APC	CXCR3-173	126511	B184803	Biolegend	
Egr2	APC	Erongr2	17-6691-82	2272709	Invitrogen	100
Eomes	PE-e610	Dan11mag	61-4875-82	2262349	Invitrogen	200
F4/80	PE-Cy5	BM8	123112	B330622	Biolegend	300
FoxP3	Pacific Blue	MF-14	126410	B339204	Biolegend	200
GATA-3	AF488	16E10A23	653808	B266231	Biolegend	100
GzmB	PerCP-Cy5.5	QA16A02	372212	B334337	Biolegend	100
I-A/I-E (MHC-II)	Pacific Blue	M5/114.15.2	107620	B308909	Biolegend	800
iNOS	AF488	CXNFT	53-5920-82	2492210	Invitrogen	100
Ki-67	BV605	16A8	652413	0341143	Biolegend	200
KLRG1	PerCP-Cy5.5	2F1/KLRG1	138417	B322568	Biolegend	200
Ly6C	PerCP-Cy5.5	HK1.4	128012	B282011	Biolegend	500
Ly6G	Spark Blue	1A8	127663	B342385	Biolegend	300
NK1.1	BV650	PK136	564143	2054280	BD	200
NKG2A	PE-Cy7	16A11	142810	B304403	Biolegend	200
OX40	BV711	OX-86	119421	B298855	Biolegend	200
PD-1	BV605	29F.1A12	135220	B333822	Biolegend	200

PD-L1	BUV737	MIH5	741877	1029626	BD	300
RORγT	PE	AFKJS-9	12-6988-82	2158265	Invitrogen	100
Siglec-F	BV711	E50-2440	740764	0346270	BD	400
Siglec-H	BV650	440c	747672	1267276	BD	200
T-bet	BV711	4B10	644819	B329082	Biolegend	100
TCF-1	APC	C63D9	37636S	1	Cell signalling technology	100
TIGIT	PE/dazzle	1G9	142110	B313921	Biolegend	400
Tim-3	BV785	RMT3-23	119725	B318350	Biolegend	300
XCR1	PE	ZET	148204	B320699	Biolegend	100
