# Supplementary material for: T-cell stimulating vaccines empower CD3 bispecific antibody therapy in solid tumors
Source: Nat Commun. 2024 Jan 2;15:48. doi: 10.1038/s41467-023-44308-6 (PMC10761684; doi:10.1038/s41467-023-44308-6)
Supplement: Supplementary file 3 — Description of Additional Supplementary Files [file 41467_2023_44308_MOESM3_ESM.pdf]

Description of Additional Supplementary Files

Supplementary Data 1. Differential gene expression for bsAb vs untreated KPC3-TRP1 tumors belonging to Figure 3. Significance was calculated used unpaired two-sided t-tests and Benjamini-Hochberg correction for multiple analysis.

Supplementary Data 2. Differential gene expression for OT-1 + OVA vacc + bsAb vs bsAb treated KPC3-TRP1 tumors belonging to Figure 3. Significance was calculated used unpaired two-sided t-tests and Benjamini-Hochberg correction for multiple analysis.

Supplementary Data 3. Differential gene expression for OT-1 + OVA vacc + bsAb vs OT-1 + OVA vacc treated KPC3-TRP1 tumors belonging to Figure 3. Significance was calculated used unpaired two-sided t-tests and Benjamini-Hochberg correction for multiple analysis.

Supplementary Data 4. Differential gene expression for OVA vacc + bsAb vs bsAb treated KPC3-TRP1 tumors belonging to Figure 6. Significance was calculated used unpaired two-sided t-tests and Benjamini-Hochberg correction for multiple analysis.
