# Supplementary material for: T-cell stimulating vaccines empower CD3 bispecific antibody therapy in solid tumors
Source: Nat Commun. 2024 Jan 2;15:48. doi: 10.1038/s41467-023-44308-6 (PMC10761684; doi:10.1038/s41467-023-44308-6)
Supplement: Supplementary file 8 — Reporting Summary [file 41467_2023_44308_MOESM8_ESM.pdf]

nature portfolio

Corresponding author(s): Thorbald van Hall

Last updated by author(s): Nov 29, 2023

Reporting Summary

Nature Portfolio wishes to improve the reproducibility of the work that we publish. This form provides structure for consistency and transparency in reporting. For further information on Nature Portfolio policies, see our Editorial Policies and the Editorial Policy Checklist.

Statistics

For all statistical analyses, confirm that the following items are present in the figure legend, table legend, main text, or Methods section.

n/a	Confirmed	
☐	☒	The exact sample size (n) for each experimental group/condition, given as a discrete number and unit of measurement
☐	☒	A statement on whether measurements were taken from distinct samples or whether the same sample was measured repeatedly
☐	☒	The statistical test(s) used AND whether they are one- or two-sided<br>*Only common tests should be described solely by name; describe more complex techniques in the Methods section.*
☒	☐	A description of all covariates tested
☐	☒	A description of any assumptions or corrections, such as tests of normality and adjustment for multiple comparisons
☐	☒	A full description of the statistical parameters including central tendency (e.g. means) or other basic estimates (e.g. regression coefficient) AND variation (e.g. standard deviation) or associated estimates of uncertainty (e.g. confidence intervals)
☐	☒	For null hypothesis testing, the test statistic (e.g. F, t, r) with confidence intervals, effect sizes, degrees of freedom and P value noted<br>*Give P values as exact values whenever suitable.*
☒	☐	For Bayesian analysis, information on the choice of priors and Markov chain Monte Carlo settings
☒	☐	For hierarchical and complex designs, identification of the appropriate level for tests and full reporting of outcomes
☒	☐	Estimates of effect sizes (e.g. Cohen's d, Pearson's r), indicating how they were calculated

Our web collection on statistics for biologists contains articles on many of the points above.

Software and code

Policy information about availability of computer code

Data collection	Bioluminescence IVIS:<br>- LivingImage software (V4.2)
Data analysis	Nanostring Transcriptomics:<br>- nSolver Analysis Software (V4.0)<br>- nSolver Advanced Analysis module(V2.0)<br><br>Generation of graphs and statistics GraphPad Prism (V9)<br><br>IHC:<br>- HALO software<br><br>Flow Cytometry:<br>- OMIQ<br>- FlowJo (v10.8.1)<br><br>Radiodistribution:<br>- AIDA (v.4.21.033)<br>- Inveon Research Workplace software package (V4.1)

Bioluminescence IVIS:
- LivingImage software (V4.2)

For manuscripts utilizing custom algorithms or software that are central to the research but not yet described in published literature, software must be made available to editors and reviewers. We strongly encourage code deposition in a community repository (e.g. GitHub). See the Nature Portfolio guidelines for submitting code & software for further information.

Data

Policy information about availability of data

All manuscripts must include a data availability statement. This statement should provide the following information, where applicable:
- Accession codes, unique identifiers, or web links for publicly available datasets
- A description of any restrictions on data availability
- For clinical datasets or third party data, please ensure that the statement adheres to our policy

All data are available in this published article (and its supplementary information files). Source data are provided with this paper.

Research involving human participants, their data, or biological material

Policy information about studies with human participants or human data. See also policy information about sex, gender (identity/presentation), and sexual orientation and race, ethnicity and racism.

Reporting on sex and gender	This manuscript does not contain any studies with human participants.
Reporting on race, ethnicity, or other socially relevant groupings	This manuscript does not contain any studies with human participants.
Population characteristics	This manuscript does not contain any studies with human participants.
Recruitment	This manuscript does not contain any studies with human participants.
Ethics oversight	This manuscript does not contain any studies with human participants.

Note that full information on the approval of the study protocol must also be provided in the manuscript.

Field-specific reporting

Please select the one below that is the best fit for your research. If you are not sure, read the appropriate sections before making your selection.

[x] Life sciences [ ] Behavioural & social sciences [ ] Ecological, evolutionary & environmental sciences

For a reference copy of the document with all sections, see nature.com/documents/nr-reporting-summary-flat.pdf

Life sciences study design

All studies must disclose on these points even when the disclosure is negative.

Sample size	To determine sample sizes for mouse experiments, we used the power and sample size calculation software and filled in the required parameters (e.g. median survival for groups) according to our previous experience, while keeping the alpha at 0.05 and the power at 0.80.
Data exclusions	For all TME studies, we excluded tumors <3x3x3 mm (length*width*height), which was an exclusion criteria that we set beforehand and use for all TME studies.
Replication	Figure 1a-c and S1a, b and f performed 1x, not repeated because results are in line with literature and rest of the results of this manuscript.<br>Figure 1d-f and S2a performed 2x independently and replicated 2x.<br>Figure 2a-c and S3a-d replicated 3x.<br>Figure 3a-e replicated in other tumor model in this manuscript (figure S4).<br>Figure 3f-i performed 1x, main conclusions confirmed using different techniques in this manuscript (figure 3 and S4).<br>Figure 4a replicated in other tumor model.<br>Figure 4b-c replicated 1x.<br>Figure 4d-f performed 1x, main conclusions are confirmed in manuscript (figure 4a-c for 4e and figure 7 for the importance of endogenous cells in 4f).<br>Figure 5a-c replicated in other tumor model 5b and 5c are replicates of each other in different tumor model.<br>Figure 5d-e performed 1x, main conclusion is in line with the other data in figure 5.<br>Figure 5f-g replicated in other tumor model 5f and 5g are replicates of each other in different tumor model.<br>Figure 5h-j performed 1x, main conclusions are in line with main conclusions from another experiment in different tumor model (figure S7h).<br>Figure 6a-b performed 1x, main conclusions confirmed using different techniques in this manuscript (figure 5, 6, S7 and S8).<br>Figure 6c-f performed 1x, replicated in other tumor model in this manuscript (figure S8).<br>Figure 7a performed 2x independently.

Figure 7b replicated 1x.
Figure 7c-d performed 1x, main conclusions are in line with conclusions from different model (figure S10 for viral model or 7a, b and e for peptide).
Figure 7e performed 1x, main conclusions are in line with conclusions from different models (figure 7a-d).

Figure S1a, b and f performed 1x, not repeated because results are in line with literature and rest of the results of this manuscript.
Figure S2a performed 2x and replicated 2x.
Figure S3b performed 1x, main conclusion confirmed using other technique (figure S3d).
Figure S3d performed 1x, main conclusions confirmed in different experiments in this manuscript (figure 2, 3 and S3b).
Figure S4a performed 1x, main conclusions confirmed using different techniques in this manuscript (figure 3d-i).
Figure S4b performed 1x, replicated in other tumor model in this manuscript (figure 3c).
Figure S4c replicated 1x.
Figure S4d-e, performed 1x, replicated in other tumor model in this manuscript (figure 3d-e).
Figure S4f-h performed 1x, main conclusions confirmed using different techniques in this manuscript (figure 3, 5 and 6).
Figure S5 performed 1x, not repeated because of lack of differences between treatments.
Figure S6a performed 1x, replicated in other tumor model.
Figure S6d-e performed 1x, main conclusions confirmed in manuscript (figure 7 for the importance of endogenous cells).
Figure S7a replicated 1x.
Figure S7b-c performed 1x, main conclusions are in line with results in other tumor models (figure 5).
Figure S7d-h performed 1x, main conclusions of S7d-g in 1 model are confirmed in second model S7h.
Figure S8a-b performed 1x, main conclusions are confirmed in figure 5, 6, S7 and S8 using different techniques and models.
Figure S8c-d performed 1x, replicated in other tumor model in this manuscript (figure 6c-d).
Figure S8e and h performed 1x, replicated in other tumor model in this manuscript (figure 6e-f).
Figure S8f-g performed 1x, replicate each other in different tumor model.
Figure S9a and g performed 1x for this time point, we have similar findings on other time points.
Figure S9b performed 2x independently.
Figure S9c replicated 1x.
Figure S9d performed 1x, main conclusions in line with similar experiment in different viral model (figure S10) or peptide models (figure 7a-d).
Figure S9e-f performed 1x, main conclusion in line with similar experiment with different experimental setup.
Figure S10a-b performed 1x, main conclusion in line with similar experiment in different viral model (figure 7d and S9d).
Figure S10c-d performed 1x for this time point, we have similar findings on other time points.

Randomization

If randomisation based on tumor size was not possible, we distributed the treatments evenly over the cages by hand before the start of the experiment, thereby attempting to disperse the treatments as much as possible to minimize cage effects. We did not have to control for any other covariates as all the mice had the same supplier, sex, age and housing conditions.

Blinding

For all experiments, different treatments were intermingled in the same cages of mice. For TME studies, researchers were blinded for the treatment groups during the data analysis. For survival studies, no additional blinding was present as tumors grew progressively or were completely controlled giving black and white outcomes.

Reporting for specific materials, systems and methods

We require information from authors about some types of materials, experimental systems and methods used in many studies. Here, indicate whether each material, system or method listed is relevant to your study. If you are not sure if a list item applies to your research, read the appropriate section before selecting a response.

Materials & experimental systems

n/a	Involved in the study	
☐	☒	Antibodies
☐	☒	Eukaryotic cell lines
☒	☐	Palaeontology and archaeology
☐	☒	Animals and other organisms
☒	☐	Clinical data
☒	☐	Dual use research of concern
☒	☐	Plants

Methods

n/a	Involved in the study	
☒	☐	ChIP-seq
☐	☒	Flow cytometry
☒	☐	MRI-based neuroimaging

Antibodies

Antibodies used

Bispecific antibody: 2C11xTA99 clone, produced by Genmab (no catalog or lot number)

IHC antibodies:
Rat anti-mouse CD8, clone 4SM15, supplier Thermo Fisher, cat. number 14-0808-80, lot number 2470230
Rabbit anti-rat IgG, clone, supplier Abcam, cat. number Ab6733, lot number GR3287864-11

Flow cytometry antibodies (in table view in supplementary methods):

Marker Fluorochrome Clone Catalog number Lot number Supplier Fold dilution
4-1BB APC 17B5 106110 B315095 Biolegend 200
Arginase 1 PE-Cy7 A1exF5 25-3697-82 2250751 Invitrogen 200
CD3 FITC 145-2C11 11-0031-85 4346374 Invitrogen 800
CD3 PE-Cy5 145-2C11 100310 B311089 Biolegend 500
CD4 BUV496 RM4-4 741051 0345594 BD 600
CD8 BUV395 53-6.7 563786 1207296 BD 400
CD8 BV711 53-6.7 100759 B332209 Biolegend 800
CD11b BUV563 M1/70 741242 0251102 BD 1200
CD11c BV605 HL3 563057 1084985 BD 200
CD19 Spark Blue 6D5 115566 B332505 Biolegend 800
CD25 BV421 PC61 102033 B284919 Biolegend 200
CD27 BV785 LG.3A10 124241 B321647 Biolegend 200
CD28 PE-Cy7 37.51 102126 B298200 Biolegend 200
CD39 PE Duha59 143803 B291343 Biolegend 200
CD44 BV510 IM-7 103043 B333218 Biolegend 600
CD44 BV785 IM-7 103059 B346798 Biolegend 100
CD45 AF700 30-F11 103128 B327672 Biolegend 400
CD45.1 APC A20 110714 B254042 Biolegend 400
CD49a BUV737 Ha31/8 741776 1162852 BD 200
CD62L BUV805 MEL-14 741924 1032098 BD 800
CD62L BV421 MEL-14 104436 B356899 Biolegend 100
CD69 BUV737 H1.2F3 612793 1159821 BD 200
CD86 BUV496 PO3 750437 0353290 BD 200
CD115 PE/dazzle AFS98 135528 B301555 Biolegend 100
CD122 PE-Cy5 TM-β1 123220 B294548 Biolegend 300
CTLA-4 BV421 UC10-4B9 106311 B378406 Biolegend 200
CXCR3 APC CXCR3-173 126511 B184803 Biolegend
Egr2 APC Erongr2 17-6691-82 2272709 Invitrogen 100
Eomes PE-e610 Dan11mag 61-4875-82 2262349 Invitrogen 200
F4/80 PE-Cy5 BM8 123112 B330622 Biolegend 300
FoxP3 Pacific Blue MF-14 126410 B339204 Biolegend 200
GATA-3 AF488 16E10A23 653808 B266231 Biolegend 100
GzmB PerCP-Cy5.5 QA16A02 372212 B334337 Biolegend 100
I-A/I-E (MHC-II) Pacific Blue M5/114.15.2 107620 B308909 Biolegend 800
iNOS AF488 CXNFT 53-5920-82 2492210 Invitrogen 100
Ki-67 BV605 16A8 652413 0341143 Biolegend 200
KLRG1 PerCP-Cy5.5 2F1/KLRG1 138417 B322568 Biolegend 200
Ly6C PerCP-Cy5.5 HK1.4 128012 B282011 Biolegend 500
Ly6G Spark Blue 1A8 127663 B342385 Biolegend 300
NK1.1 BV650 PK136 564143 2054280 BD 200
NKG2A PE-Cy7 16A11 142810 B304403 Biolegend 200
OX40 BV711 OX-86 119421 B298855 Biolegend 200
PD-1 BV605 29F.1A12 135220 B333822 Biolegend 200
PD-L1 BUV737 MIH5 741877 1029626 BD 300
RORγT PE AFKJS-9 12-6988-82 2158265 Invitrogen 100
Siglec-F BV711 E50-2440 740764 0346270 BD 400
Siglec-H BV650 440c 747672 1267276 BD 200
T-bet BV711 4B10 644819 B329082 Biolegend 100
TCF-1 APC C63D9 37636S 1 Cell signalling technology 100
TIGIT PE/dazzle 1G9 142110 B313921 Biolegend 400
Tim-3 BV785 RMT3-23 119725 B318350 Biolegend 300
XCR1 PE ZET 148204 B320699 Biolegend 100

Validation

The website of the FACS and IHC antibody suppliers show statements of validation for all of the FACS and IHC antibodies.
The bispecific antibody has been validated in two previous papers that are cited in the manuscript as well (Labrijn et al., Scientific Reports, 2017; Benonnisson et al., Mol. Cancer. Ther., 2019)

Eukaryotic cell lines

Policy information about [cell lines and Sex and Gender in Research]

Cell line source(s)

The B16F10 murine melanoma cell line was purchased from the ATCC (CRL-6475)
The KPC3 cell line was obtained from the lab of David Tuveson (Hingorani et al., Cancer Cell, 2005)
The MC38 cell line was obtained from the lab of Ferry Ossendorp (Schrörs et al., Frontiers in Immunology, 2023)
The KPC3-TRP1 and MC38-TRP1 cell lines were generated in house from the KPC3 or MC38 cell lines as described for KPC3-TRP1 in the methods section (Benonnison et al., Mol. Cancer Ther., 2019)

Authentication	The B16F10, KPC3 and MC38 cell lines were authenticated using short tandem repeat markers by IDEXX BioAnalytics
Mycoplasma contamination	All cell lines tested negative for mycoplasma contamination
Commonly misidentified lines (See ICLAC register)	We used no commonly misidentified cell lines

Animals and other research organisms

Policy information about studies involving animals; ARRIVE guidelines recommended for reporting animal research, and Sex and Gender in Research

Laboratory animals	We used 8 weeks old C57BL/6, CXCR3 KO (Jackson Laboratories, 005796), OT-1 (Jackson Laboratories, 003831), Albino C57BL/6 (Jackson Laboratories, 000058) and homebred OT-1xTbiLuc mice. Mice were housed in the following conditions: dark/light cycle 06.30-07.00 sunrise, 07.00-18.00 day time, 18.00-18.30 sunset, 18.30-06.30 night time; 20-22 degrees Celsius; 50-60% humidity.
Wild animals	This study did not involve wild animals
Reporting on sex	This study only involves data obtained from male mice, as the B16 tumor is derived from a male mouse and we wanted to prevent the occurrence of any unwanted immune responses and remain consistent throughout the manuscript.
Field-collected samples	This study did not involve samples collected from the field
Ethics oversight	All mouse studies were approved by the Dutch animal ethics committee (CCD) and the local Animal Welfare Body of the LUMC or Radboud UMC on the permit numbers AVD116002015271 and AVD11600202010004, or AVD1030020209645 respectively.

Note that full information on the approval of the study protocol must also be provided in the manuscript.

Plants

Seed stocks	This manuscript does not contain any studies performed with plants
Novel plant genotypes	This manuscript does not contain any studies performed with plants
Authentication	This manuscript does not contain any studies performed with plants

Flow Cytometry

Plots

Confirm that:

- [ ] The axis labels state the marker and fluorochrome used (e.g. CD4-FITC).
- [ ] The axis scales are clearly visible. Include numbers along axes only for bottom left plot of group (a 'group' is an analysis of identical markers).
- [x] All plots are contour plots with outliers or pseudocolor plots.
- [x] A numerical value for number of cells or percentage (with statistics) is provided.

Methodology

Sample preparation	Tumors, spleens and blood were harvested from mice. Single cell suspensions from KPC3 and KPC3-TRP1 tumors were prepared by physical fragmentation followed by 10 minutes incubation with 2.5 mg/mL liberase TM (Roche) at 37°C in a humidified atmosphere containing 5% CO2. Then, cell suspensions from KPC3 and KPC3-TRP1 tumors, or whole B16F10 tumors and spleens were minced through a 70 µm cell strainer (Falcon). For blood and spleen samples, red blood cells were lysed for 3 minutes with lysis buffer (pharmacy in house) at room temperature.
Instrument	The experiments were measured on an LSRFortessa cytometer (BD), or Aurora 5L spectral flow cytometer (Cytek)
Software	The data was analyzed with FlowJo v10.8.1 (Treestar), or OMIQ software
Cell population abundance	We did not use cell sorting for this study
Gating strategy	Lymphoid cells were gating with the following strategy:

Cells were gated based on FSC/SSC (leaving out the very small cell fragments and bigger cell aggregates)
Single cells were gated based on FSC-H and FSC-A (leaving out cells that are not nicely in the straight diagonal)
Live cells were gated based on FSC-A and Viability dye (leaving out cells that stained positive for viability dye)
CD45+ cells were gated based on FSC-A and CD45 (leaving out all cells that had no expression of CD45)
CD3+ cells were gated based on CD3 and CD8 (leaving out all cells that were negative for CD3)
CD4+ cells were gated based on CD4 and CD8 (leaving out all cells that were not only positive for CD4)
FoxP3+ cells were gated based on FoxP3 and CD4 (leaving out all cells that were not positive for FoxP3)
CD8+ cells were gated based on CD4 and CD8 (leaving out all cells that were not only positive for CD8)
OT-1 cells were gated based on CD8 and CD45.1 (leaving our all cells that were not positive for CD45.1)
NK cells were gated from CD3- cells based on NK1.1 and CD19 (excluding all cells that were not single positive for NK1.1)
B cells were gated from CD3- cells based on NK1.1 and CD19 (excluding all cells that were not single positive for CD19)

Myeloid cells were gated with the following strategy:
Cells were gated based on FSC/SSC (leaving out the very small cell fragments and bigger cell aggregates)
Single cells were gated based on FSC-H and FSC-A (leaving out cells that are not nicely in the straight diagonal)
Live cells were gated based on FSC-A and Viability dye (leaving out cells that stained positive for viability dye)
CD45+ cells were gated based on FSC-A and CD45 (leaving out all cells that had no expression of CD45)
CD11b-/CD11c+ cells were gated from CD45+ cells based on CD11b and CD11c (leaving out all cells that were CD11b+ or negative for both CD11b and CD11c)
pDCs were gated from CD11b-/CD11c+ cells based on Siglec-H and CD11c (leaving out all cells that were negative for Siglec-H)
cDC1s were gated from Siglec-H- cells based on MHC-II and CD11c (leaving out all cells with low CD11c, or no MHCII expression)
CD11b+ cells were gated from CD45+ cells based on CD11b and CD11c (leaving out all cells negative for CD11b)
Neutrophils were gated from CD11b+ cells based on Ly6G and CD11b (leaving out all cells low in Ly6G or CD11b)
Eosinophils were gated from Ly6G- cells based on Siglec-F and SSC-A (leaving out all cells without Siglec-F expression or low SSC-A)
Macrophages were gated from Siglec-F- cells based on F4/80 and CD11b (leaving out all cells with low F4/80 or CD11b expression)
CD11b+/CD11c+ cells were gated from the remaining CD11b+ cells after macrophages were taken out based on CD11b and CD11c (leaving out all cells negative for CD11c)
cDC2s were gated from CD11b+/CD11c+ cells based on MHC-II and Ly6C expression (retaining all cells positive for MHC-II and negative for Ly6C)
moDCs were gated from CD11b+/CD11c+ cells based on MHC-II and Ly6C expression (retaining all cells double-positive for MHC-II and Ly6C

☒ Tick this box to confirm that a figure exemplifying the gating strategy is provided in the Supplementary Information.
