# Supplementary material for: T-cell stimulating vaccines empower CD3 bispecific antibody therapy in solid tumors
Source: Nat Commun. 2024 Jan 2;15:48. doi: 10.1038/s41467-023-44308-6 (PMC10761684; doi:10.1038/s41467-023-44308-6)
Supplement: Supplementary file 2 — Peer Review File [file 41467_2023_44308_MOESM2_ESM.pdf]

nature portfolio

Peer Review File

T-cell stimulating vaccines empower CD3 bispecific antibody therapy in solid tumors

Open Access This file is licensed under a Creative Commons Attribution 4.0 International License, which permits use, sharing, adaptation, distribution and reproduction in any medium or format, as long as you give appropriate credit to the original author(s) and the source, provide a link to the Creative Commons license, and indicate if changes were made. In the cases where the authors are anonymous, such as is the case for the reports of anonymous peer reviewers, author attribution should be to 'Anonymous Referee' followed by a clear attribution to the source work. The images or other third party material in this file are included in the article's Creative Commons license, unless indicated otherwise in a credit line to the material. If material is not included in the article's Creative Commons license and your intended use is not permitted by statutory regulation or exceeds the permitted use, you will need to obtain permission directly from the copyright holder. To view a copy of this license, visit http://creativecommons.org/licenses/by/4.0/.

Editorial Note: This manuscript has been previously reviewed at another journal that is not operating a transparent peer review scheme. This document only contains reviewer comments and rebuttal letters for versions considered at *Nature Communications*.

REVIEWERS' COMMENTS

Reviewer #2 (Remarks to the Author):

The authors present impressive and thoughtfully designed experimental data demonstrating the potentiation of bispecific Ab (bsAb) therapy for solid tumors partly as follow-up to their excellent manuscript [Hreinn Benonisson et al., Mol Can Ther. 2019].

The work is technically impressive including dual-color bioluminescent tracking of T cell trafficking and activation state as well as bsAb radio-isotope labeling to assess pharmacokinetic alterations in their experimental models.

Reviewer #2 (Remarks to the Author):

The authors present impressive and thoughtfully designed experimental data demonstrating the potentiation of bispecific Ab (bsAb) therapy for solid tumors partly as follow-up to their excellent manuscript [Hreinn Benonisson et al., Mol Can Ther. 2019].
The work is technically impressive including dual-color bioluminescent tracking of T cell trafficking and activation state as well as bsAb radio-isotope labeling to assess pharmacokinetic alterations in their experimental models.

Thank you.
